# Supplementary figures and images for: A comprehensive in silico analysis of the deleterious nonsynonymous SNPs of human FOXP2 protein
Source: PLoS One. 2022 Aug 9;17(8):e0272625. doi: 10.1371/journal.pone.0272625 (PMC9362936; doi:10.1371/journal.pone.0272625)

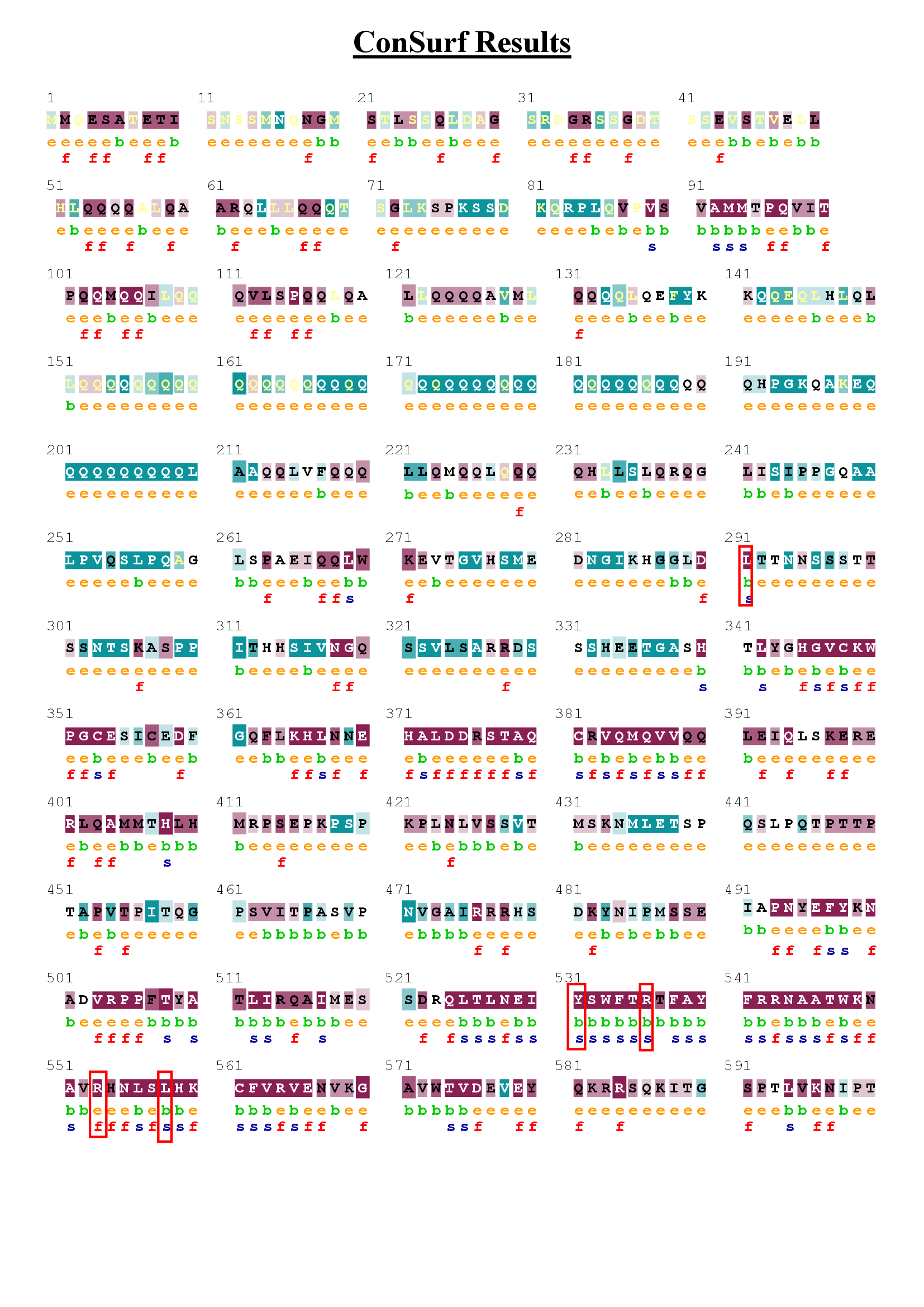

Supplement: S1 Fig — (TIFF) [file pone.0272625.s001.tiff]
