# Supplementary material for: A comprehensive in silico analysis of the deleterious nonsynonymous SNPs of human FOXP2 protein
Source: PLoS One. 2022 Aug 9;17(8):e0272625. doi: 10.1371/journal.pone.0272625 (PMC9362936; doi:10.1371/journal.pone.0272625)
Supplement: S1 Table — (DOCX) [file pone.0272625.s002.docx]

**The list of reference sequence ID, alteration of allele, protein accession no, position of amino acid and residual change of all 393 non-synonymous SNPs**

| **Serial no.** | **rs ID** | **Alteration of Allele** | **Protein accession no** | **Position of amino acid** | **Residue change** |
| --- | --- | --- | --- | --- | --- |
|  | rs111801240 | Q [CAA] > H [CAT] | NP_055306.1 | 177 | Q (Gln) > H (His) |
|  | rs121908377 | R [CGT] > H [CAT] | NP_055306.1 | 553 | R (Arg) > H (His) |
|  | [rs140766407](https://www.ncbi.nlm.nih.gov/snp/rs140766407) | L [CTG] > P [CCG] | NP_055306.1 | 714 | L (Leu) > P (Pro) |
|  | [rs145154396](https://www.ncbi.nlm.nih.gov/snp/rs145154396) | N [AAT] > S [AGT] | NP_055306.1 | 646 | N (Asn) > S (Ser) |
|  | [rs147624408](https://www.ncbi.nlm.nih.gov/snp/rs147624408) | E [GAA] > D [GAT] | NP_055306.1 | 700 | E (Glu) > D (Asp) |
|  | [rs182138317](https://www.ncbi.nlm.nih.gov/snp/rs182138317) | A [GCC] > T [ACC] | NP_055306.1 | 615 | A (Ala) > T (Thr) |
|  | rs201084683 | N [AAT] > D [GAT] | NP_055306.1 | 646 | N (Asn) > D (Asp) |
|  | [rs201320940](https://www.ncbi.nlm.nih.gov/snp/rs201320940) | S [TCA] > A [GCA] | NP_055306.1 | 488 | S (Ser) > A (Ala) |
|  | [rs201649896](https://www.ncbi.nlm.nih.gov/snp/rs201649896) | Q [CAA] > L [CTA] | NP_055306.1 | 17 | Q (Gln) > L (Leu) |
|  | [rs376460299](https://www.ncbi.nlm.nih.gov/snp/rs376460299) | H [CAC] > Q [CAG] | NP_055306.1 | 408 | H (His) > Q (Gln) |
|  | [rs529427719](https://www.ncbi.nlm.nih.gov/snp/rs529427719) | G [GGC] > D [GAC] | NP_055306.1 | 661 | G (Gly) > D (Asp) |
|  | [rs564204446](https://www.ncbi.nlm.nih.gov/snp/rs564204446) | Q [CAA] > K [AAA] | NP_055306.1 | 173 | Q (Gln) > K (Lys) |
|  | [rs727503937](https://www.ncbi.nlm.nih.gov/snp/rs727503937) | S [AGT] > I [ATT] | NP_055306.1 | 428 | S (Ser) > I (Ile) |
|  | [rs757373725](https://www.ncbi.nlm.nih.gov/snp/rs757373725) | S [AGC] > N [AAC] | NP_055306.1 | 619 | S (Ser) > N (Asn) |
|  | [rs759256511](https://www.ncbi.nlm.nih.gov/snp/rs759256511) | R [CGA] > Q [CAA] | NP_055306.1 | 328 | R (Arg) > Q (Gln) |
|  | [rs759949520](https://www.ncbi.nlm.nih.gov/snp/rs759949520) | Q [CAG] > K [AAG] | NP_055306.1 | 67 | Q (Gln) > K (Lys) |
|  | [rs763263115](https://www.ncbi.nlm.nih.gov/snp/rs763263115) | N [AAT] > D [GAT] | NP_055306.1 | 18 | N (Asn) > D (Asp) |
|  | [rs766476648](https://www.ncbi.nlm.nih.gov/snp/rs766476648) | N [AAT] > H [CAT] | NP_055306.1 | 597 | N (Asn) > H (His) |
|  | [rs797045587](https://www.ncbi.nlm.nih.gov/snp/rs797045587) | M [ATG] > I [ATT] | NP_055306.1 | 94 | M (Met) > I (Ile) |
|  | [rs879253772](https://www.ncbi.nlm.nih.gov/snp/rs879253772) | Y [TAC] > H [CAC] | NP_055306.1 | 531 | Y (Tyr) > H (His) |
|  | [rs886061916](https://www.ncbi.nlm.nih.gov/snp/rs886061916) | S [AGC] > C [TGC] | NP_055306.1 | 330 | S (Ser) > C (Cys) |
|  | [rs1563064505](https://www.ncbi.nlm.nih.gov/snp/rs1563064505) | T [ACC] > I [ATC] | NP_055306.1 | 448 | T (Thr) > I (Ile) |
|  | [rs1563067518](https://www.ncbi.nlm.nih.gov/snp/rs1563067518) | LV [TTAGTA] > FI [TTTATA] | NP_055306.1 | 594 | LV (LeuVal) > FI (PheIle) |
|  | [rs1584969604](https://www.ncbi.nlm.nih.gov/snp/rs1584969604) | Q [CAG] > R [CGG] | NP_055306.1 | 179 | Q (Gln) > R (Arg) |
|  | [rs74507296](https://www.ncbi.nlm.nih.gov/snp/rs74507296) | Q [CAG] > P [CCG] | NP_055306.1 | 97 | Q (Gln) > P (Pro) |
|  | [rs112732214](https://www.ncbi.nlm.nih.gov/snp/rs112732214) | L [CTG] > P [CCG] | NP_055306.1 | 558 | L (Leu) > P (Pro) |
|  | [rs138374374](https://www.ncbi.nlm.nih.gov/snp/rs138374374) | S [TCG] > A [GCG] | NP_055306.1 | 298 | S (Ser) > A (Ala) |
|  | [rs141431326](https://www.ncbi.nlm.nih.gov/snp/rs141431326) | G [GGA] > R [AGA] | NP_055306.1 | 361 | G (Gly) > R (Arg) |
|  | [rs142199218](https://www.ncbi.nlm.nih.gov/snp/rs142199218) | M [ATG] > V [GTG] | NP_055306.1 | 487 | M (Met) > V (Val) |
|  | rs143019903 | Q [CAA] > H [CAT] | NP_055306.1 | 225 | Q (Gln) > H (His) |
|  | rs145254341 | P [CCG] > R [CGG] | NP_055306.1 | 666 | P (Pro) > R (Arg) |
|  | rs148201242 | L [CTC] > I [ATC] | NP_055306.1 | 241 | L (Leu) > I (Ile) |
|  | rs149805525 | T [ACC] > N [AAC] | NP_055306.1 | 430 | T (Thr) > N (Asn) |
|  | rs150907165 | S [TCC] > C [TGC] | NP_055306.1 | 297 | S (Ser) > C (Cys) |
|  | rs181670107 | S [AGC] > G [GGC] | NP_055306.1 | 24 | S (Ser) > G (Gly) |
|  | rs185960561 | T [ACC] > I [ATC] | NP_055306.1 | 40 | T (Thr) > I (Ile) |
|  | rs189863975 | A [GCC] > V [GTC] | NP_055306.1 | 452 | A (Ala) > V (Val) |
|  | rs199776572 | T [ACG] > M [ATG] | NP_055306.1 | 451 | T (Thr) > M (Met) |
|  | [rs202008325](https://www.ncbi.nlm.nih.gov/snp/rs202008325) | M [ATG] > V [GTG] | NP_055306.1 | 687 | M (Met) > V (Val) |
|  | rs368203434 | A [GCA] > E [GAA] | NP_055306.1 | 211 | A (Ala) > E (Glu) |
|  | rs369313543 | P [CCC] > T [ACC] | NP_055306.1 | 416 | P (Pro) > T (Thr) |
|  | rs369839969 | D [GAT] > E [GAA] | NP_055306.1 | 713 | D (Asp) > E (Glu) |
|  | rs372477540 | Q [CAA] > R [CGA] | NP_055306.1 | 445 | Q (Gln) > R (Arg) |
|  | rs372704196 | H [CAT] > R [CGT] | NP_055306.1 | 333 | H (His) > R (Arg) |
|  | rs375163729 | S [TCT] > A [GCT] | NP_055306.1 | 321 | S (Ser) > A (Ala) |
|  | rs377217556 | I [ATT] > T [ACT] | NP_055306.1 | 652 | I (Ile) > T (Thr) |
|  | rs377372067 | T [ACA] > I [ATA] | NP_055306.1 | 7 | T (Thr) > I (Ile) |
|  | rs377420314 | Y [TAT] > C [TGT] | NP_055306.1 | 604 | Y (Tyr) > C (Cys) |
|  | rs377588856 | S [AGC] > T [ACC] | NP_055306.1 | 45 | S (Ser) > T (Thr) |
|  | rs531504911 | S [TCG] > P [CCG] | NP_055306.1 | 255 | S (Ser) > P (Pro) |
|  | rs541820585 | T [ACC] > N [AAC] | NP_055306.1 | 407 | T (Thr) > N (Asn) |
|  | rs555128980 | T [ACT] > I [ATT] | NP_055306.1 | 304 | T (Thr) > I (Ile) |
|  | rs562313396 | P [CCG] > L [CTG] | NP_055306.1 | 660 | P (Pro) > L (Leu) |
|  | rs563897082 | I [ATA] > V [GTA] | NP_055306.1 | 316 | I (Ile) > V (Val) |
|  | rs565909014 | I [ATA] > M [ATG] | NP_055306.1 | 598 | I (Ile) > M (Met) |
|  | rs566961630 | R [CGT] > C [TGT] | NP_055306.1 | 553 | R (Arg) > C (Cys) |
|  | rs576347421 | E [GAA] > K [AAA] | NP_055306.1 | 643 | E (Glu) > K (Lys) |
|  | rs576347421 | E [GAA] > Q [CAA] | NP_055306.1 | 643 | E (Glu) > Q (Gln) |
|  | rs745342916 | D [GAC] > G [GGC] | NP_055306.1 | 375 | D (Asp) > G (Gly) |
|  | [rs745618497](https://www.ncbi.nlm.nih.gov/snp/rs745618497) | L [CTA] > R [CGA] | NP_055306.1 | 23 | L (Leu) > R (Arg) |
|  | [rs745987986](https://www.ncbi.nlm.nih.gov/snp/rs745987986) | V [GTC] > I [ATC] | NP_055306.1 | 641 | V (Val) > I (Ile) |
|  | [rs746400429](https://www.ncbi.nlm.nih.gov/snp/rs746400429) | L [CTG] > V [GTG] | NP_055306.1 | 714 | L (Leu) > V (Val) |
|  | [rs746524111](https://www.ncbi.nlm.nih.gov/snp/rs746524111) | S [AGC] > N [AAC] | NP_055306.1 | 21 | S (Ser) > N (Asn) |
|  | [rs746703454](https://www.ncbi.nlm.nih.gov/snp/rs746703454) | L [CTT] > F [TTT] | NP_055306.1 | 108 | L (Leu) > F (Phe) |
|  | [rs746884663](https://www.ncbi.nlm.nih.gov/snp/rs746884663) | I [ATA] > V [GTA] | NP_055306.1 | 630 | I (Ile) > V (Val) |
|  | [rs747155760](https://www.ncbi.nlm.nih.gov/snp/rs747155760) | A [GCA] > S [TCA] | NP_055306.1 | 307 | A (Ala) > S (Ser) |
|  | [rs747467925](https://www.ncbi.nlm.nih.gov/snp/rs747467925) | V [GTC] > I [ATC] | NP_055306.1 | 673 | V (Val) > I (Ile) |
|  | [rs748073993](https://www.ncbi.nlm.nih.gov/snp/rs748073993) | A [GCT] > T [ACT] | NP_055306.1 | 57 | A (Ala) > T (Thr) |
|  | [rs748231233](https://www.ncbi.nlm.nih.gov/snp/rs748231233) | D [GAC] > N [AAC] | NP_055306.1 | 281 | D (Asp) > N (Asn) |
|  | [rs748314629](https://www.ncbi.nlm.nih.gov/snp/rs748314629) | D [GAC] > N [AAC] | NP_055306.1 | 702 | D (Asp) > N (Asn) |
|  | [rs748597582](https://www.ncbi.nlm.nih.gov/snp/rs748597582) | F [TTT] > L [TTG] | NP_055306.1 | 360 | F (Phe) > L (Leu) |
|  | [rs748878832](https://www.ncbi.nlm.nih.gov/snp/rs748878832) | E [GAA] > G [GGA] | NP_055306.1 | 392 | E (Glu) > G (Gly) |
|  | [rs749254802](https://www.ncbi.nlm.nih.gov/snp/rs749254802) | Q [CAG] > H [CAC] | NP_055306.1 | 220 | Q (Gln) > H (His) |
|  | [rs749278394](https://www.ncbi.nlm.nih.gov/snp/rs749278394) | P [CCA] > S [TCA] | NP_055306.1 | 453 | P (Pro) > S (Ser) |
|  | [rs749932113](https://www.ncbi.nlm.nih.gov/snp/rs749932113) | S [AGT] > R [AGG] | NP_055306.1 | 79 | S (Ser) > R (Arg) |
|  | [rs750028450](https://www.ncbi.nlm.nih.gov/snp/rs750028450) | A [GCT] > V [GTT] | NP_055306.1 | 264 | A (Ala) > V (Val) |
|  | [rs750287608](https://www.ncbi.nlm.nih.gov/snp/rs750287608) | L [TTA] > S [TCA] | NP_055306.1 | 689 | L (Leu) > S (Ser) |
|  | [rs750505654](https://www.ncbi.nlm.nih.gov/snp/rs750505654) | V [GTG] > M [ATG] | NP_055306.1 | 98 | V (Val) > M (Met) |
|  | [rs750582959](https://www.ncbi.nlm.nih.gov/snp/rs750582959) | T [ACT] > S [TCT] | NP_055306.1 | 336 | T (Thr) > S (Ser) |
|  | [rs750701057](https://www.ncbi.nlm.nih.gov/snp/rs750701057) | S [TCA] > P [CCA] | NP_055306.1 | 36 | S (Ser) > P (Pro) |
|  | [rs750736305](https://www.ncbi.nlm.nih.gov/snp/rs750736305) | S [AGT] > G [GGT] | NP_055306.1 | 13 | S (Ser) > G (Gly) |
|  | [rs751166815](https://www.ncbi.nlm.nih.gov/snp/rs751166815) | T [ACA] > I [ATA] | NP_055306.1 | 438 | T (Thr) > I (Ile) |
|  | [rs751732210](https://www.ncbi.nlm.nih.gov/snp/rs751732210) | A [GCT] > S [TCT] | NP_055306.1 | 539 | A (Ala) > S (Ser) |
|  | [rs751931499](https://www.ncbi.nlm.nih.gov/snp/rs751931499) | P [CCT] > T [ACT] | NP_055306.1 | 422 | P (Pro) > T (Thr) |
|  | [rs752426529](https://www.ncbi.nlm.nih.gov/snp/rs752426529) | S [AGT] > N [AAT] | NP_055306.1 | 620 | S (Ser) > N (Asn) |
|  | [rs752528443](https://www.ncbi.nlm.nih.gov/snp/rs752528443) | T [ACC] > P [CCC] | NP_055306.1 | 300 | T (Thr) > P (Pro) |
|  | [rs753356750](https://www.ncbi.nlm.nih.gov/snp/rs753356750) | N [AAC] > K [AAG] | NP_055306.1 | 657 | N (Asn) > K (Lys) |
|  | [rs753394697](https://www.ncbi.nlm.nih.gov/snp/rs753394697) | S [AGT] > R [AGG] | NP_055306.1 | 325 | S (Ser) > R (Arg) |
|  | [rs753640569](https://www.ncbi.nlm.nih.gov/snp/rs753640569) | E [GAA] > D [GAC] | NP_055306.1 | 272 | E (Glu) > D (Asp) |
|  | [rs753643915](https://www.ncbi.nlm.nih.gov/snp/rs753643915) | S [TCG] > L [TTG] | NP_055306.1 | 298 | S (Ser) > L (Leu) |
|  | [rs753658194](https://www.ncbi.nlm.nih.gov/snp/rs753658194) | P [CCC] > S [TCC] | NP_055306.1 | 486 | P (Pro) > S (Ser) |
|  | [rs753732678](https://www.ncbi.nlm.nih.gov/snp/rs753732678) | P [CCA] > T [ACA] | NP_055306.1 | 493 | P (Pro) > T (Thr) |
|  | [rs753857220](https://www.ncbi.nlm.nih.gov/snp/rs753857220) | E [GAA] > K [AAA] | NP_055306.1 | 700 | E (Glu) > K (Lys) |
|  | [rs754271344](https://www.ncbi.nlm.nih.gov/snp/rs754271344) | S [TCT] > C [TGT] | NP_055306.1 | 42 | S (Ser) > C (Cys) |
|  | [rs754361203](https://www.ncbi.nlm.nih.gov/snp/rs754361203) | Q [CAG] > R [CGG] | NP_055306.1 | 239 | Q (Gln) > R (Arg) |
|  | [rs754927033](https://www.ncbi.nlm.nih.gov/snp/rs754927033) | G [GGA] > E [GAA] | NP_055306.1 | 275 | G (Gly) > E (Glu) |
|  | [rs755297474](https://www.ncbi.nlm.nih.gov/snp/rs755297474) | Y [TAT] > C [TGT] | NP_055306.1 | 343 | Y (Tyr) > C (Cys) |
|  | [rs755668650](https://www.ncbi.nlm.nih.gov/snp/rs755668650) | Q [CAG] > H [CAC] | NP_055306.1 | 82 | Q (Gln) > H (His) |
|  | [rs755825043](https://www.ncbi.nlm.nih.gov/snp/rs755825043) | D [GAC] > N [AAC] | NP_055306.1 | 481 | D (Asp) > N (Asn) |
|  | [rs755829229](https://www.ncbi.nlm.nih.gov/snp/rs755829229) | Q [CAG] > H [CAT] | NP_055306.1 | 231 | Q (Gln) > H (His) |
|  | [rs755981069](https://www.ncbi.nlm.nih.gov/snp/rs755981069) | V [GTG] > G [GGG] | NP_055306.1 | 690 | V (Val) > G (Gly) |
|  | [rs756019068](https://www.ncbi.nlm.nih.gov/snp/rs756019068) | T [ACC] > N [AAC] | NP_055306.1 | 446 | T (Thr) > N (Asn) |
|  | [rs756184095](https://www.ncbi.nlm.nih.gov/snp/rs756184095) | V [GTG] > G [GGG] | NP_055306.1 | 98 | V (Val) > G (Gly) |
|  | [rs756555227](https://www.ncbi.nlm.nih.gov/snp/rs756555227) | S [AGT] > N [AAT] | NP_055306.1 | 13 | S (Ser) > N (Asn) |
|  | [rs757255710](https://www.ncbi.nlm.nih.gov/snp/rs757255710) | T [ACT] > S [TCT] | NP_055306.1 | 95 | T (Thr) > S (Ser) |
|  | [rs757270050](https://www.ncbi.nlm.nih.gov/snp/rs757270050) | E [GAG] > V [GTG] | NP_055306.1 | 334 | E (Glu) > V (Val) |
|  | [rs757351650](https://www.ncbi.nlm.nih.gov/snp/rs757351650) | T [ACA] > P [CCA] | NP_055306.1 | 7 | T (Thr) > P (Pro) |
|  | [rs758052155](https://www.ncbi.nlm.nih.gov/snp/rs758052155) | C [TGC] > W [TGG] | NP_055306.1 | 662 | C (Cys) > W (Trp) |
|  | [rs758080021](https://www.ncbi.nlm.nih.gov/snp/rs758080021) | P [CCT] > L [CTT] | NP_055306.1 | 627 | P (Pro) > L (Leu) |
|  | [rs758423327](https://www.ncbi.nlm.nih.gov/snp/rs758423327) | T [ACT] > P [CCT] | NP_055306.1 | 304 | T (Thr) > P (Pro) |
|  | [rs758427088](https://www.ncbi.nlm.nih.gov/snp/rs758427088) | R [CGG] > Q [CAG] | NP_055306.1 | 536 | R (Arg) > Q (Gln) |
|  | [rs758513311](https://www.ncbi.nlm.nih.gov/snp/rs758513311) | P [CCT] > L [CTT] | NP_055306.1 | 505 | P (Pro) > L (Leu) |
|  | [rs758714987](https://www.ncbi.nlm.nih.gov/snp/rs758714987) | I [ATT] > V [GTT] | NP_055306.1 | 705 | I (Ile) > V (Val) |
|  | [rs760114813](https://www.ncbi.nlm.nih.gov/snp/rs760114813) | V [GTG] > A [GCG] | NP_055306.1 | 472 | V (Val) > A (Ala) |
|  | [rs760226272](https://www.ncbi.nlm.nih.gov/snp/rs760226272) | V [GTT] > I [ATT] | NP_055306.1 | 323 | V (Val) > I (Ile) |
|  | [rs760596736](https://www.ncbi.nlm.nih.gov/snp/rs760596736) | H [CAC] > D [GAC] | NP_055306.1 | 408 | H (His) > D (Asp) |
|  | [rs760842249](https://www.ncbi.nlm.nih.gov/snp/rs760842249) | G [GGT] > D [GAT] | NP_055306.1 | 38 | G (Gly) > D (Asp) |
|  | [rs761170451](https://www.ncbi.nlm.nih.gov/snp/rs761170451) | T [ACC] > P [CCC] | NP_055306.1 | 465 | T (Thr) > P (Pro) |
|  | [rs761174159](https://www.ncbi.nlm.nih.gov/snp/rs761174159) | R [CGT] > S [AGT] | NP_055306.1 | 238 | R (Arg) > S (Ser) |
|  | [rs761262072](https://www.ncbi.nlm.nih.gov/snp/rs761262072) | V [GTG] > L [TTG] | NP_055306.1 | 426 | V (Val) > L (Leu) |
|  | [rs762007110](https://www.ncbi.nlm.nih.gov/snp/rs762007110) | S [AGC] > N [AAC] | NP_055306.1 | 31 | S (Ser) > N (Asn) |
|  | [rs762414429](https://www.ncbi.nlm.nih.gov/snp/rs762414429) | M [ATG] > V [GTG] | NP_055306.1 | 431 | M (Met) > V (Val) |
|  | [rs762564041](https://www.ncbi.nlm.nih.gov/snp/rs762564041) | R [CGT] > C [TGT] | NP_055306.1 | 401 | R (Arg) > C (Cys) |
|  | [rs762699659](https://www.ncbi.nlm.nih.gov/snp/rs762699659) | S [TCA] > P [CCA] | NP_055306.1 | 90 | S (Ser) > P (Pro) |
|  | [rs762817913](https://www.ncbi.nlm.nih.gov/snp/rs762817913) | D [GAT] > A [GCT] | NP_055306.1 | 28 | D (Asp) > A (Ala) |
|  | [rs763246182](https://www.ncbi.nlm.nih.gov/snp/rs763246182) | D [GAC] > H [CAC] | NP_055306.1 | 644 | D (Asp) > H (His) |
|  | [rs763302537](https://www.ncbi.nlm.nih.gov/snp/rs763302537) | I [ATA] > T [ACA] | NP_055306.1 | 316 | I (Ile) > T (Thr) |
|  | [rs763449275](https://www.ncbi.nlm.nih.gov/snp/rs763449275) | S [TCT] > F [TTT] | NP_055306.1 | 75 | S (Ser) > F (Phe) |
|  | [rs763457983](https://www.ncbi.nlm.nih.gov/snp/rs763457983) | A [GCC] > T [ACC] | NP_055306.1 | 474 | A (Ala) > T (Thr) |
|  | [rs763537390](https://www.ncbi.nlm.nih.gov/snp/rs763537390) | N [AAT] > K [AAG] | NP_055306.1 | 295 | N (Asn) > K (Lys) |
|  | [rs763629604](https://www.ncbi.nlm.nih.gov/snp/rs763629604) | A [GCA] > T [ACA] | NP_055306.1 | 680 | A (Ala) > T (Thr) |
|  | [rs763694805](https://www.ncbi.nlm.nih.gov/snp/rs763694805) | T [ACA] > P [CCA] | NP_055306.1 | 438 | T (Thr) > P (Pro) |
|  | [rs763694805](https://www.ncbi.nlm.nih.gov/snp/rs763694805) | T [ACA] > A [GCA] | NP_055306.1 | 438 | T (Thr) > A (Ala) |
|  | [rs764109995](https://www.ncbi.nlm.nih.gov/snp/rs764109995) | A [GCG] > V [GTG] | NP_055306.1 | 6 | A (Ala) > V (Val) |
|  | [rs764574245](https://www.ncbi.nlm.nih.gov/snp/rs764574245) | S [AGC] > N [AAC] | NP_055306.1 | 78 | S (Ser) > N (Asn) |
|  | [rs764574245](https://www.ncbi.nlm.nih.gov/snp/rs764574245) | S [AGC] > I [ATC] | NP_055306.1 | 78 | S (Ser) > I (Ile) |
|  | [rs765157455](https://www.ncbi.nlm.nih.gov/snp/rs765157455) | R [CGG] > G [GGG] | NP_055306.1 | 536 | R (Arg) > G (Gly) |
|  | rs765157455 | R [CGG] > W [TGG] | NP_055306.1 | 536 | R (Arg) > W (Trp) |
|  | [rs765574065](https://www.ncbi.nlm.nih.gov/snp/rs765574065) | Q [CAG] > K [AAG] | NP_055306.1 | 613 | Q (Gln) > K (Lys) |
|  | [rs765647678](https://www.ncbi.nlm.nih.gov/snp/rs765647678) | Q [CAG] > R [CGG] | NP_055306.1 | 67 | Q (Gln) > R (Arg) |
|  | [rs765980369](https://www.ncbi.nlm.nih.gov/snp/rs765980369) | A [GCT] > G [GGT] | NP_055306.1 | 614 | A (Ala) > G (Gly) |
|  | [rs765980960](https://www.ncbi.nlm.nih.gov/snp/rs765980960) | R [AGA] > G [GGA] | NP_055306.1 | 83 | R (Arg) > G (Gly) |
|  | [rs766020871](https://www.ncbi.nlm.nih.gov/snp/rs766020871) | A [GCC] > V [GTC] | NP_055306.1 | 492 | A (Ala) > V (Val) |
|  | [rs766803095](https://www.ncbi.nlm.nih.gov/snp/rs766803095) | S [AGC] > G [GGC] | NP_055306.1 | 41 | S (Ser) > G (Gly) |
|  | [rs766956626](https://www.ncbi.nlm.nih.gov/snp/rs766956626) | S [TCC] > T [ACC] | NP_055306.1 | 296 | S (Ser) > T (Thr) |
|  | [rs766965457](https://www.ncbi.nlm.nih.gov/snp/rs766965457) | R [CGT] > H [CAT] | NP_055306.1 | 238 | R (Arg) > H (His) |
|  | [rs767291302](https://www.ncbi.nlm.nih.gov/snp/rs767291302) | A [GCC] > T [ACC] | NP_055306.1 | 338 | A (Ala) > T (Thr) |
|  | [rs767291302](https://www.ncbi.nlm.nih.gov/snp/rs767291302) | A [GCC] > S [TCC] | NP_055306.1 | 338 | A (Ala) > S (Ser) |
|  | [rs767652094](https://www.ncbi.nlm.nih.gov/snp/rs767652094) | G [GGC] > S [AGC] | NP_055306.1 | 247 | G (Gly) > S (Ser) |
|  | [rs767652094](https://www.ncbi.nlm.nih.gov/snp/rs767652094) | G [GGC] > R [CGC] | NP_055306.1 | 247 | G (Gly) > R (Arg) |
|  | [rs768186362](https://www.ncbi.nlm.nih.gov/snp/rs768186362) | S [AGC] > N [AAC] | NP_055306.1 | 654 | S (Ser) > N (Asn) |
|  | [rs768186362](https://www.ncbi.nlm.nih.gov/snp/rs768186362) | S [AGC] > T [ACC] | NP_055306.1 | 654 | S (Ser) > T (Thr) |
|  | [rs768728605](https://www.ncbi.nlm.nih.gov/snp/rs768728605) | L [CTC] > V [GTC] | NP_055306.1 | 222 | L (Leu) > V (Val) |
|  | [rs769148228](https://www.ncbi.nlm.nih.gov/snp/rs769148228) | L [CTG] > M [ATG] | NP_055306.1 | 130 | L (Leu) > M (Met) |
|  | [rs769218331](https://www.ncbi.nlm.nih.gov/snp/rs769218331) | V [GTG] > L [CTG] | NP_055306.1 | 317 | V (Val) > L (Leu) |
|  | [rs769296657](https://www.ncbi.nlm.nih.gov/snp/rs769296657) | T [ACT] > A [GCT] | NP_055306.1 | 378 | T (Thr) > A (Ala) |
|  | [rs769641584](https://www.ncbi.nlm.nih.gov/snp/rs769641584) | V [GTA] > I [ATA] | NP_055306.1 | 565 | V (Val) > I (Ile) |
|  | [rs769729974](https://www.ncbi.nlm.nih.gov/snp/rs769729974) | A [GCG] > E [GAG] | NP_055306.1 | 197 | A (Ala) > E (Glu) |
|  | [rs769729974](https://www.ncbi.nlm.nih.gov/snp/rs769729974) | A [GCG] > G [GGG] | NP_055306.1 | 197 | A (Ala) > G (Gly) |
|  | [rs769729974](https://www.ncbi.nlm.nih.gov/snp/rs769729974) | A [GCG] > V [GTG] | NP_055306.1 | 197 | A (Ala) > V (Val) |
|  | [rs770001809](https://www.ncbi.nlm.nih.gov/snp/rs770001809) | H [CAT] > Y [TAT] | NP_055306.1 | 313 | H (His) > Y (Tyr) |
|  | [rs770093841](https://www.ncbi.nlm.nih.gov/snp/rs770093841) | M [ATG] > V [GTG] | NP_055306.1 | 15 | M (Met) > V (Val) |
|  | [rs770301569](https://www.ncbi.nlm.nih.gov/snp/rs770301569) | N [AAC] > D [GAC] | NP_055306.1 | 294 | N (Asn) > D (Asp) |
|  | [rs770756604](https://www.ncbi.nlm.nih.gov/snp/rs770756604) | Q [CAG] > P [CCG] | NP_055306.1 | 157 | Q (Gln) > P (Pro) |
|  | [rs770923990](https://www.ncbi.nlm.nih.gov/snp/rs770923990) | I [ATA] > M [ATG] | NP_055306.1 | 630 | I (Ile) > M (Met) |
|  | [rs771268489](https://www.ncbi.nlm.nih.gov/snp/rs771268489) | A [GCA] > V [GTA] | NP_055306.1 | 307 | A (Ala) > V (Val) |
|  | [rs771314598](https://www.ncbi.nlm.nih.gov/snp/rs771314598) | P [CCT] > L [CTT] | NP_055306.1 | 709 | P (Pro) > L (Leu) |
|  | [rs771438075](https://www.ncbi.nlm.nih.gov/snp/rs771438075) | P [CCA] > L [CTA] | NP_055306.1 | 466 | P (Pro) > L (Leu) |
|  | [rs771840840](https://www.ncbi.nlm.nih.gov/snp/rs771840840) | Q [CAG] > R [CGG] | NP_055306.1 | 183 | Q (Gln) > R (Arg) |
|  | [rs772009456](https://www.ncbi.nlm.nih.gov/snp/rs772009456) | I [ATT] > V [GTT] | NP_055306.1 | 284 | I (Ile) > V (Val) |
|  | [rs772294351](https://www.ncbi.nlm.nih.gov/snp/rs772294351) | S [AGT] > N [AAT] | NP_055306.1 | 71 | S (Ser) > N (Asn) |
|  | [rs772694863](https://www.ncbi.nlm.nih.gov/snp/rs772694863) | G [GGA] > E [GAA] | NP_055306.1 | 319 | G (Gly) > E (Glu) |
|  | [rs772881522](https://www.ncbi.nlm.nih.gov/snp/rs772881522) | F [TTT] > L [CTT] | NP_055306.1 | 138 | F (Phe) > L (Leu) |
|  | [rs773700650](https://www.ncbi.nlm.nih.gov/snp/rs773700650) | I [ATC] > T [ACC] | NP_055306.1 | 464 | I (Ile) > T (Thr) |
|  | [rs773759127](https://www.ncbi.nlm.nih.gov/snp/rs773759127) | Q [CAG] > H [CAC] | NP_055306.1 | 117 | Q (Gln) > H (His) |
|  | [rs773874222](https://www.ncbi.nlm.nih.gov/snp/rs773874222) | P [CCT] > L [CTT] | NP_055306.1 | 76 | P (Pro) > L (Leu) |
|  | [rs773947565](https://www.ncbi.nlm.nih.gov/snp/rs773947565) | D [GAC] > V [GTC] | NP_055306.1 | 684 | D (Asp) > V (Val) |
|  | [rs774292922](https://www.ncbi.nlm.nih.gov/snp/rs774292922) | Q [CAG] > R [CGG] | NP_055306.1 | 581 | Q (Gln) > R (Arg) |
|  | [rs774304280](https://www.ncbi.nlm.nih.gov/snp/rs774304280) | M [ATG] > I [ATC] | NP_055306.1 | 224 | M (Met) > I (Ile) |
|  | [rs774408585](https://www.ncbi.nlm.nih.gov/snp/rs774408585) | S [AGC] > G [GGC] | NP_055306.1 | 31 | S (Ser) > G (Gly) |
|  | [rs774842735](https://www.ncbi.nlm.nih.gov/snp/rs774842735) | V [GTG] > M [ATG] | NP_055306.1 | 678 | V (Val) > M (Met) |
|  | [rs774842735](https://www.ncbi.nlm.nih.gov/snp/rs774842735) | V [GTG] > M [ATG] | NP_055306.1 | 678 | V (Val) > L (Leu) |
|  | [rs775655580](https://www.ncbi.nlm.nih.gov/snp/rs775655580) | Q [CAG] > R [CGG] | NP_055306.1 | 86 | Q (Gln) > R (Arg) |
|  | [rs775730461](https://www.ncbi.nlm.nih.gov/snp/rs775730461) | T [ACA] > A [GCA] | NP_055306.1 | 70 | T (Thr) > A (Ala) |
|  | [rs775743904](https://www.ncbi.nlm.nih.gov/snp/rs775743904) | S [TCT] > P [CCT] | NP_055306.1 | 419 | S (Ser) > P (Pro) |
|  | [rs775819986](https://www.ncbi.nlm.nih.gov/snp/rs775819986) | N [AAT] > S [AGT] | NP_055306.1 | 295 | N (Asn) > S (Ser) |
|  | [rs776670223](https://www.ncbi.nlm.nih.gov/snp/rs776670223) | Q [CAG] > H [CAT] | NP_055306.1 | 160 | Q (Gln) > H (His) |
|  | [rs776679391](https://www.ncbi.nlm.nih.gov/snp/rs776679391) | M [ATG] > L [CTG] | NP_055306.1 | 411 | M (Met) > L (Leu) |
|  | [rs776679391](https://www.ncbi.nlm.nih.gov/snp/rs776679391) | M [ATG] > V [GTG] | NP_055306.1 | 411 | M (Met) > V (Val) |
|  | [rs776966569](https://www.ncbi.nlm.nih.gov/snp/rs776966569) | A [GCC] > T [ACC] | NP_055306.1 | 467 | A (Ala) > T (Thr) |
|  | [rs777042566](https://www.ncbi.nlm.nih.gov/snp/rs777042566) | L [CTT] > I [ATT] | NP_055306.1 | 65 | L (Leu) > I (Ile) |
|  | [rs777132475](https://www.ncbi.nlm.nih.gov/snp/rs777132475) | T [ACT] > I [ATT] | NP_055306.1 | 292 | T (Thr) > I (Ile) |
|  | [rs777361904](https://www.ncbi.nlm.nih.gov/snp/rs777361904) | M [ATG] > V [GTG] | NP_055306.1 | 20 | M (Met) > V (Val) |
|  | [rs777560011](https://www.ncbi.nlm.nih.gov/snp/rs777560011) | G [GGA] > V [GTA] | NP_055306.1 | 628 | G (Gly) > V (Val) |
|  | [rs778026488](https://www.ncbi.nlm.nih.gov/snp/rs778026488) | K [AAA] > Q [CAA] | NP_055306.1 | 285 | K (Lys) > Q (Gln) |
|  | [rs778396336](https://www.ncbi.nlm.nih.gov/snp/rs778396336) | A [GCA] > T [ACA] | NP_055306.1 | 372 | A (Ala) > T (Thr) |
|  | [rs778396336](https://www.ncbi.nlm.nih.gov/snp/rs778396336) | A [GCA] > S [TCA] | NP_055306.1 | 372 | A (Ala) > S (Ser) |
|  | [rs778748061](https://www.ncbi.nlm.nih.gov/snp/rs778748061) | R [CGC] > C [TGC] | NP_055306.1 | 399 | R (Arg) > C (Cys) |
|  | [rs778767619](https://www.ncbi.nlm.nih.gov/snp/rs778767619) | M [ATG] > V [GTG] | NP_055306.1 | 279 | M (Met) > V (Val) |
|  | [rs779157210](https://www.ncbi.nlm.nih.gov/snp/rs779157210) | P [CCT] > S [TCT] | NP_055306.1 | 627 | P (Pro) > S (Ser) |
|  | [rs779632352](https://www.ncbi.nlm.nih.gov/snp/rs779632352) | E [GAA] > K [AAA] | NP_055306.1 | 48 | E (Glu) > K (Lys) |
|  | [rs779754644](https://www.ncbi.nlm.nih.gov/snp/rs779754644) | W [TGG] > R [CGG] | NP_055306.1 | 270 | W (Trp) > R (Arg) |
|  | [rs779921362](https://www.ncbi.nlm.nih.gov/snp/rs779921362) | Y [TAC] > C [TGC] | NP_055306.1 | 483 | Y (Tyr) > C (Cys) |
|  | [rs780212480](https://www.ncbi.nlm.nih.gov/snp/rs780212480) | T [ACC] > I [ATC] | NP_055306.1 | 100 | T (Thr) > I (Ile) |
|  | [rs780371962](https://www.ncbi.nlm.nih.gov/snp/rs780371962) | T [ACT] > A [GCT] | NP_055306.1 | 341 | T (Thr) > A (Ala) |
|  | [rs780676420](https://www.ncbi.nlm.nih.gov/snp/rs780676420) | T [ACT] > S [TCT] | NP_055306.1 | 292 | T (Thr) > S (Ser) |
|  | [rs780869585](https://www.ncbi.nlm.nih.gov/snp/rs780869585) | A [GCA] > T [ACA] | NP_055306.1 | 250 | A (Ala) > T (Thr) |
|  | [rs781437567](https://www.ncbi.nlm.nih.gov/snp/rs781437567) | Q [CAA] > H [CAT] | NP_055306.1 | 173 | Q (Gln) > H (His) |
|  | [rs866570059](https://www.ncbi.nlm.nih.gov/snp/rs866570059) | S [TCC] > F [TTC] | NP_055306.1 | 296 | S (Ser) > F (Phe) |
|  | [rs866894515](https://www.ncbi.nlm.nih.gov/snp/rs866894515) | H [CAC] > Y [TAC] | NP_055306.1 | 672 | H (His) > Y (Tyr) |
|  | [rs867418322](https://www.ncbi.nlm.nih.gov/snp/rs867418322) | S [TCC] > F [TTC] | NP_055306.1 | 305 | S (Ser) > F (Phe) |
|  | [rs868366293](https://www.ncbi.nlm.nih.gov/snp/rs868366293) | A [GCC] > T [ACC] | NP_055306.1 | 92 | A (Ala) > T (Thr) |
|  | [rs879771053](https://www.ncbi.nlm.nih.gov/snp/rs879771053) | G [GGA] > E [GAA] | NP_055306.1 | 460 | G (Gly) > E (Glu) |
|  | [rs889178260](https://www.ncbi.nlm.nih.gov/snp/rs889178260) | S [TCA] > L [TTA] | NP_055306.1 | 308 | S (Ser) > L (Leu) |
|  | [rs889341368](https://www.ncbi.nlm.nih.gov/snp/rs889341368) | E [GAG] > K [AAG] | NP_055306.1 | 676 | E (Glu) > K (Lys) |
|  | [rs895017794](https://www.ncbi.nlm.nih.gov/snp/rs895017794) | S [AGT] > G [GGT] | NP_055306.1 | 428 | S (Ser) > G (Gly) |
|  | [s899776651](https://www.ncbi.nlm.nih.gov/snp/rs899776651) | G [GGA] > A [GCA] | NP_055306.1 | 194 | G (Gly) > A (Ala) |
|  | [rs907456099](https://www.ncbi.nlm.nih.gov/snp/rs907456099) | A [GCA] > E [GAA] | NP_055306.1 | 60 | A (Ala) > E (Glu) |
|  | [rs908446199](https://www.ncbi.nlm.nih.gov/snp/rs908446199) | A [GCT] > V [GTT] | NP_055306.1 | 127 | A (Ala) > V (Val) |
|  | [rs909392122](https://www.ncbi.nlm.nih.gov/snp/rs909392122) | G [GGG] > R [AGG] | NP_055306.1 | 288 | G (Gly) > R (Arg) |
|  | [rs915879314](https://www.ncbi.nlm.nih.gov/snp/rs915879314) | A [GCA] > V [GTA] | NP_055306.1 | 617 | A (Ala) > V (Val) |
|  | [rs920208430](https://www.ncbi.nlm.nih.gov/snp/rs920208430) | L [TTA] > S [TCA] | NP_055306.1 | 710 | L (Leu) > S (Ser) |
|  | [rs928369081](https://www.ncbi.nlm.nih.gov/snp/rs928369081) | H [CAC] > P [CCC] | NP_055306.1 | 695 | H (His) > P (Pro) |
|  | [rs937208031](https://www.ncbi.nlm.nih.gov/snp/rs937208031) | N [AAC] > K [AAA] | NP_055306.1 | 294 | N (Asn) > K (Lys) |
|  | [rs938443435](https://www.ncbi.nlm.nih.gov/snp/rs938443435) | H [CAC] > Q [CAG] | NP_055306.1 | 695 | H (His) > Q (Gln) |
|  | [rs940890058](https://www.ncbi.nlm.nih.gov/snp/rs940890058) | D [GAC] > N [AAC] | NP_055306.1 | 290 | D (Asp) > N (Asn) |
|  | [rs942638508](https://www.ncbi.nlm.nih.gov/snp/rs942638508) | S [TCT] > P [CCT] | NP_055306.1 | 42 | S (Ser) > P (Pro) |
|  | [rs942769559](https://www.ncbi.nlm.nih.gov/snp/rs942769559) | Q [CAG] > E [GAG] | NP_055306.1 | 164 | Q (Gln) > E (Glu) |
|  | [rs944127085](https://www.ncbi.nlm.nih.gov/snp/rs944127085) | S [TCT] > Y [TAT] | NP_055306.1 | 321 | S (Ser) > Y (Tyr) |
|  | [rs945804979](https://www.ncbi.nlm.nih.gov/snp/rs945804979) | T [ACT] > I [ATT] | NP_055306.1 | 336 | T (Thr) > I (Ile) |
|  | [rs948249504](https://www.ncbi.nlm.nih.gov/snp/rs948249504) | R [AGA] > G [GGA] | NP_055306.1 | 703 | R (Arg) > G (Gly) |
|  | [rs952693445](https://www.ncbi.nlm.nih.gov/snp/rs952693445) | I [ATA] > T [ACA] | NP_055306.1 | 630 | I (Ile) > T (Thr) |
|  | [rs955135535](https://www.ncbi.nlm.nih.gov/snp/rs952693445) | G [GGA] > R [AGA] | NP_055306.1 | 473 | G (Gly) > R (Arg) |
|  | [rs959136398](https://www.ncbi.nlm.nih.gov/snp/rs959136398) | Q [CAG] > E [GAG] | NP_055306.1 | 180 | Q (Gln) > E (Glu) |
|  | [rs960171629](https://www.ncbi.nlm.nih.gov/snp/rs960171629) | D [GAT] > H [CAT] | NP_055306.1 | 650 | D (Asp) > H (His) |
|  | [rs960747265](https://www.ncbi.nlm.nih.gov/snp/rs960747265) | S [TCT] > F [TTT] | NP_055306.1 | 711 | S (Ser) > F (Phe) |
|  | [rs976182523](https://www.ncbi.nlm.nih.gov/snp/rs976182523) | Q [CAA] > R [CGA] | NP_055306.1 | 226 | Q (Gln) > R (Arg) |
|  | [rs985325734](https://www.ncbi.nlm.nih.gov/snp/rs985325734) | G [GGC] > V [GTC] | NP_055306.1 | 283 | G (Gly) > V (Val) |
|  | [rs1003245699](https://www.ncbi.nlm.nih.gov/snp/rs1003245699) | Q [CAG] > E [GAG] | NP_055306.1 | 179 | Q (Gln) > E (Glu) |
|  | [rs1010843580](https://www.ncbi.nlm.nih.gov/snp/rs1010843580) | Q [CAA] > K [AAA] | NP_055306.1 | 171 | Q (Gln) > K (Lys) |
|  | [rs1023491570](https://www.ncbi.nlm.nih.gov/snp/rs1023491570) | E [GAA] > G [GGA] | NP_055306.1 | 715 | E (Glu) > G (Gly) |
|  | [rs1029480822](https://www.ncbi.nlm.nih.gov/snp/rs1029480822) | Q [CAG] > L [CTG] | NP_055306.1 | 185 | Q (Gln) > L (Leu) |
|  | [rs1038896563](https://www.ncbi.nlm.nih.gov/snp/rs1038896563) | Q [CAG] > K [AAG] | NP_055306.1 | 167 | Q (Gln) > K (Lys) |
|  | rs1039811463 | S [TCA] > A [GCA] | NP_055306.1 | 322 | S (Ser) > A (Ala) |
|  | [rs1046073933](https://www.ncbi.nlm.nih.gov/snp/rs1046073933) | M [ATG] > I [ATT] | NP_055306.1 | 431 | M (Met) > I (Ile) |
|  | [rs1050432134](https://www.ncbi.nlm.nih.gov/snp/rs1050432134) | S [TCC] > C [TGC] | NP_055306.1 | 302 | S (Ser) > C (Cys) |
|  | [rs1054712701](https://www.ncbi.nlm.nih.gov/snp/rs1054712701) | S [TCG] > W [TGG] | NP_055306.1 | 255 | S (Ser) > W (Trp) |
|  | [rs1054712701](https://www.ncbi.nlm.nih.gov/snp/rs1054712701) | S [TCG] > L [TTG] | NP_055306.1 | 255 | S (Ser) > L (Leu) |
|  | [rs1055108779](https://www.ncbi.nlm.nih.gov/snp/rs1055108779) | Q [CAG] > E [GAG] | NP_055306.1 | 158 | Q (Gln) > E (Glu) |
|  | [rs1056005508](https://www.ncbi.nlm.nih.gov/snp/rs1056005508) | S [AGC] > G [GGC] | NP_055306.1 | 78 | S (Ser) > G (Gly) |
|  | [rs1056692528](https://www.ncbi.nlm.nih.gov/snp/rs1056692528) | V [GTG] > A [GCG] | NP_055306.1 | 426 | V (Val) > A (Ala) |
|  | [rs1158252110](https://www.ncbi.nlm.nih.gov/snp/rs1158252110) | Q [CAG] > R [CGG] | NP_055306.1 | 218 | Q (Gln) > R (Arg) |
|  | [rs1158865993](https://www.ncbi.nlm.nih.gov/snp/rs1158865993) | R [CGT] > H [CAT] | NP_055306.1 | 543 | R (Arg) > H (His) |
|  | [rs1164029715](https://www.ncbi.nlm.nih.gov/snp/rs1164029715) | S [AGT] > N [AAT] | NP_055306.1 | 37 | S (Ser) > N (Asn) |
|  | [rs1168362217](https://www.ncbi.nlm.nih.gov/snp/rs1168362217) | Q [CAG] > H [CAC] | NP_055306.1 | 97 | Q (Gln) > H (His) |
|  | [rs1169006435](https://www.ncbi.nlm.nih.gov/snp/rs1169006435) | L [TTA] > I [ATA] | NP_055306.1 | 27 | L (Leu) > I (Ile) |
|  | [rs1175210435](https://www.ncbi.nlm.nih.gov/snp/rs1175210435) | L [CTA] > P [CCA] | NP_055306.1 | 289 | L (Leu) > P (Pro) |
|  | [rs1177378764](https://www.ncbi.nlm.nih.gov/snp/rs1177378764) | A [GCA] > S [TCA] | NP_055306.1 | 326 | A (Ala) > S (Ser) |
|  | \| [rs1183578823](https://www.ncbi.nlm.nih.gov/snp/rs1183578823) \| \| --- \| | G [GGA] > V [GTA] | NP_055306.1 | 570 | G (Gly) > V (Val) |
|  | [rs1184039801](https://www.ncbi.nlm.nih.gov/snp/rs1184039801) | P [CCA] > L [CTA] | NP_055306.1 | 697 | P (Pro) > L (Leu) |
|  | [rs1188410790](https://www.ncbi.nlm.nih.gov/snp/rs1188410790) | Q [CAG] > P [CCG] | NP_055306.1 | 3 | Q (Gln) > P (Pro) |
|  | [rs1190852721](https://www.ncbi.nlm.nih.gov/snp/rs1190852721) | T [ACC] > P [CCC] | NP_055306.1 | 446 | T (Thr) > P (Pro) |
|  | [rs1191637371](https://www.ncbi.nlm.nih.gov/snp/rs1191637371) | P [CCT] > S [TCT] | NP_055306.1 | 115 | P (Pro) > S (Ser) |
|  | [rs1192209213](https://www.ncbi.nlm.nih.gov/snp/rs1192209213) | G [GGC] > S [AGC] | NP_055306.1 | 283 | G (Gly) > S (Ser) |
|  | [rs1202904573](https://www.ncbi.nlm.nih.gov/snp/rs1202904573) | E [GAA] > G [GGA] | NP_055306.1 | 48 | E (Glu) > G (Gly) |
|  | [rs1207268336](https://www.ncbi.nlm.nih.gov/snp/rs1207268336) | V [GTT] > L [CTT] | NP_055306.1 | 276 | V (Val) > L (Leu) |
|  | [rs1212829863](https://www.ncbi.nlm.nih.gov/snp/rs1212829863) | E [GAA] > D [GAT] | NP_055306.1 | 712 | E (Glu) > D (Asp) |
|  | [rs1215855483](https://www.ncbi.nlm.nih.gov/snp/rs1215855483) | S [TCC] > F [TTC] | NP_055306.1 | 439 | S (Ser) > F (Phe) |
|  | [rs1218149104](https://www.ncbi.nlm.nih.gov/snp/rs1218149104) | I [ATA] > L [CTA] | NP_055306.1 | 513 | I (Ile) > L (Leu) |
|  | [rs1219589831](https://www.ncbi.nlm.nih.gov/snp/rs1219589831) | R [CGA] > L [CTA] | NP_055306.1 | 376 | R (Arg) > L (Leu) |
|  | [rs1221297848](https://www.ncbi.nlm.nih.gov/snp/rs1221297848) | K [AAG] > M [ATG] | NP_055306.1 | 195 | K (Lys) > M (Met) |
|  | [rs1224001486](https://www.ncbi.nlm.nih.gov/snp/rs1224001486) | Q [CAG] > H [CAC] | NP_055306.1 | 441 | Q (Gln) > H (His) |
|  | [rs1224336230](https://www.ncbi.nlm.nih.gov/snp/rs1224336230) | W [TGG] > C [TGT] | NP_055306.1 | 270 | W (Trp) > C (Cys) |
|  | [rs1225170530](https://www.ncbi.nlm.nih.gov/snp/rs1225170530) | I [ATT] > T [ACT] | NP_055306.1 | 679 | I (Ile) > T (Thr) |
|  | [rs1227082843](https://www.ncbi.nlm.nih.gov/snp/rs1227082843) | P [CCA] > S [TCA] | NP_055306.1 | 310 | P (Pro) > S (Ser) |
|  | [rs1230115579](https://www.ncbi.nlm.nih.gov/snp/rs1230115579) | V [GTG] > M [ATG] | NP_055306.1 | 469 | V (Val) > M (Met) |
|  | [rs1230202192](https://www.ncbi.nlm.nih.gov/snp/rs1230202192) | L [CTG] > P [CCG] | NP_055306.1 | 638 | L (Leu) > P (Pro) |
|  | [rs1236343176](https://www.ncbi.nlm.nih.gov/snp/rs1236343176) | P [CCT] > T [ACT] | NP_055306.1 | 709 | P (Pro) > T (Thr) |
|  | [rs1237536573](https://www.ncbi.nlm.nih.gov/snp/rs1237536573) | S [AGT] > R [CGT] | NP_055306.1 | 71 | S (Ser) > R (Arg) |
|  | [rs1240394269](https://www.ncbi.nlm.nih.gov/snp/rs1237536573) | I [ATA] > V [GTA] | NP_055306.1 | 475 | I (Ile) > V (Val) |
|  | [rs1243599633](https://www.ncbi.nlm.nih.gov/snp/rs1243599633) | Q [CAA] > L [CTA] | NP_055306.1 | 178 | Q (Gln) > L (Leu) |
|  | [rs1244944146](https://www.ncbi.nlm.nih.gov/snp/rs1244944146) | E [GAA] > K [AAA] | NP_055306.1 | 43 | E (Glu) > K (Lys) |
|  | [rs1250616703](https://www.ncbi.nlm.nih.gov/snp/rs1250616703) | N [AAT] > I [ATT] | NP_055306.1 | 567 | N (Asn) > I (Ile) |
|  | [rs1251042963](https://www.ncbi.nlm.nih.gov/snp/rs1251042963) | N [AAT] > Y [TAT] | NP_055306.1 | 694 | N (Asn) > Y (Tyr) |
|  | [rs1254975807](https://www.ncbi.nlm.nih.gov/snp/rs1254975807) | V [GTA] > A [GCA] | NP_055306.1 | 47 | V (Val) > A (Ala) |
|  | [rs1256659385](https://www.ncbi.nlm.nih.gov/snp/rs1256659385) | I [ATC] > L [CTC] | NP_055306.1 | 464 | I (Ile) > L (Leu) |
|  | [rs1269264458](https://www.ncbi.nlm.nih.gov/snp/rs1269264458) | L [TTA] > V [GTA] | NP_055306.1 | 594 | L (Leu) > V (Val) |
|  | [rs1270486405](https://www.ncbi.nlm.nih.gov/snp/rs1270486405) | R [CGA] > Q [CAA] | NP_055306.1 | 412 | R (Arg) > Q (Gln) |
|  | [rs1270889010](https://www.ncbi.nlm.nih.gov/snp/rs1270889010) | V [GTT] > A [GCT] | NP_055306.1 | 563 | V (Val) > A (Ala) |
|  | [rs1273852421](https://www.ncbi.nlm.nih.gov/snp/rs1273852421) | N [AAC] > S [AGC] | NP_055306.1 | 12 | N (Asn) > S (Ser) |
|  | [rs1275101579](https://www.ncbi.nlm.nih.gov/snp/rs1275101579) | M [ATG] > T [ACG] | NP_055306.1 | 431 | M (Met) > T (Thr) |
|  | [rs1276375707](https://www.ncbi.nlm.nih.gov/snp/rs1276375707) | L [TTA] > F [TTT] | NP_055306.1 | 269 | L (Leu) > F (Phe) |
|  | [rs1276471970](https://www.ncbi.nlm.nih.gov/snp/rs1276471970) | E [GAA] > K [AAA] | NP_055306.1 | 400 | E (Glu) > K (Lys) |
|  | [rs1277076789](https://www.ncbi.nlm.nih.gov/snp/rs1277076789) | N [AAT] > K [AAA] | NP_055306.1 | 471 | N (Asn) > K (Lys) |
|  | [rs1277744858](https://www.ncbi.nlm.nih.gov/snp/rs1277744858) | T [ACC] > I [ATC] | NP_055306.1 | 300 | T (Thr) > I (Ile) |
|  | [rs1278315350](https://www.ncbi.nlm.nih.gov/snp/rs1278315350) | H [CAT] > R [CGT] | NP_055306.1 | 669 | H (His) > R (Arg) |
|  | [rs1280238984](https://www.ncbi.nlm.nih.gov/snp/rs1280238984) | A [GCC] > T [ACC] | NP_055306.1 | 492 | A (Ala) > T (Thr) |
|  | [rs1281884581](https://www.ncbi.nlm.nih.gov/snp/rs1281884581) | Q [CAG] > H [CAC] | NP_055306.1 | 200 | Q (Gln) > H (His) |
|  | [rs1283187808](https://www.ncbi.nlm.nih.gov/snp/rs1283187808) | V [GTA] > G [GGA] | NP_055306.1 | 44 | V (Val) > G (Gly) |
|  | [rs1285369301](https://www.ncbi.nlm.nih.gov/snp/rs1285369301) | P [CCA] > S [TCA] | NP_055306.1 | 440 | P (Pro) > S (Ser) |
|  | [rs1290398957](https://www.ncbi.nlm.nih.gov/snp/rs1290398957) | P [CCC] > R [CGC] | NP_055306.1 | 416 | P (Pro) > R (Arg) |
|  | [rs1290752827](https://www.ncbi.nlm.nih.gov/snp/rs1290752827) | P [CCC] > S [TCC] | NP_055306.1 | 470 | P (Pro) > S (Ser) |
|  | [rs1291795029](https://www.ncbi.nlm.nih.gov/snp/rs1291795029) | G [GGC] > V [GTC] | NP_055306.1 | 636 | G (Gly) > V (Val) |
|  | [rs1294048735](https://www.ncbi.nlm.nih.gov/snp/rs1294048735) | S [TCA] > T [ACA] | NP_055306.1 | 663 | S (Ser) > T (Thr) |
|  | [rs1295112601](https://www.ncbi.nlm.nih.gov/snp/rs1295112601) | H [CAT] > R [CGT] | NP_055306.1 | 345 | H (His) > R (Arg) |
|  | [rs1300216902](https://www.ncbi.nlm.nih.gov/snp/rs1300216902) | N [AAC] > S [AGC] | NP_055306.1 | 494 | N (Asn) > S (Ser) |
|  | [rs1302127838](https://www.ncbi.nlm.nih.gov/snp/rs1302127838) | N [AAT] > K [AAG] | NP_055306.1 | 424 | N (Asn) > K (Lys) |
|  | [rs1308003272](https://www.ncbi.nlm.nih.gov/snp/rs1308003272) | V [GTG] > A [GCG] | NP_055306.1 | 317 | V (Val) > A (Ala) |
|  | [rs1308658577](https://www.ncbi.nlm.nih.gov/snp/rs1308658577) | S [TCT] > F [TTT] | NP_055306.1 | 521 | S (Ser) > F (Phe) |
|  | [rs1315572414](https://www.ncbi.nlm.nih.gov/snp/rs1315572414) | G [GGG] > E [GAG] | NP_055306.1 | 337 | G (Gly) > E (Glu) |
|  | [rs1323738523](https://www.ncbi.nlm.nih.gov/snp/rs1323738523) | I [ATT] > L [CTT] | NP_055306.1 | 457 | I (Ile) > L (Leu) |
|  | [rs1330279241](https://www.ncbi.nlm.nih.gov/snp/rs1330279241) | V [GTG] > M [ATG] | NP_055306.1 | 89 | V (Val) > M (Met) |
|  | [rs1330529378](https://www.ncbi.nlm.nih.gov/snp/rs1330529378) | H [CAC] > Y [TAC] | NP_055306.1 | 340 | H (His) > Y (Tyr) |
|  | [rs1331241429](https://www.ncbi.nlm.nih.gov/snp/rs1331241429) | L [CTT] > F [TTT] | NP_055306.1 | 236 | L (Leu) > F (Phe) |
|  | [rs1338895971](https://www.ncbi.nlm.nih.gov/snp/rs1338895971) | Q [CAG] > E [GAG] | NP_055306.1 | 155 | Q (Gln) > E (Glu) |
|  | [rs1339945518](https://www.ncbi.nlm.nih.gov/snp/rs1339945518) | Q [CAG] > H [CAT] | NP_055306.1 | 213 | Q (Gln) > H (His) |
|  | [rs1342506252](https://www.ncbi.nlm.nih.gov/snp/rs1342506252) | V [GTG] > M [ATG] | NP_055306.1 | 87 | V (Val) > M (Met) |
|  | [rs1343377230](https://www.ncbi.nlm.nih.gov/snp/rs1343377230) | M [ATG] > K [AAG] | NP_055306.1 | 406 | M (Met) > K (Lys) |
|  | [rs1345935751](https://www.ncbi.nlm.nih.gov/snp/rs1345935751) | N [AAT] > S [AGT] | NP_055306.1 | 471 | N (Asn) > S (Ser) |
|  | [rs1347015690](https://www.ncbi.nlm.nih.gov/snp/rs1347015690) | T [ACC] > S [AGC] | NP_055306.1 | 600 | T (Thr) > S (Ser) |
|  | [rs1347150082](https://www.ncbi.nlm.nih.gov/snp/rs1347150082) | I [ATC] > F [TTC] | NP_055306.1 | 99 | I (Ile) > F (Phe) |
|  | [rs1347299046](https://www.ncbi.nlm.nih.gov/snp/rs1347299046) | Q [CAA] > E [GAA] | NP_055306.1 | 196 | Q (Gln) > E (Glu) |
|  | [rs1348548231](https://www.ncbi.nlm.nih.gov/snp/rs1348548231) | S [AGT] > N [AAT] | NP_055306.1 | 468 | S (Ser) > N (Asn) |
|  | [rs1349469230](https://www.ncbi.nlm.nih.gov/snp/rs1349469230) | S [TCC] > T [ACC] | NP_055306.1 | 688 | S (Ser) > T (Thr) |
|  | [rs1355061824](https://www.ncbi.nlm.nih.gov/snp/rs1355061824) | Q [CAG] > R [CGG] | NP_055306.1 | 665 | Q (Gln) > R (Arg) |
|  | [rs1357512652](https://www.ncbi.nlm.nih.gov/snp/rs1357512652) | Q [CAA] > H [CAT] | NP_055306.1 | 63 | Q (Gln) > H (His) |
|  | [rs1359238420](https://www.ncbi.nlm.nih.gov/snp/rs1359238420) | Q [CAG] > P [CCG] | NP_055306.1 | 182 | Q (Gln) > P (Pro) |
|  | [rs1359401489](https://www.ncbi.nlm.nih.gov/snp/rs1359401489) | S [TCC] > T [ACC] | NP_055306.1 | 315 | S (Ser) > T (Thr) |
|  | [rs1362466494](https://www.ncbi.nlm.nih.gov/snp/rs1362466494) | P [CCA] > S [TCA] | NP_055306.1 | 677 | P (Pro) > S (Ser) |
|  | [rs1362984312](https://www.ncbi.nlm.nih.gov/snp/rs1362984312) | I [ATA] > V [GTA] | NP_055306.1 | 311 | I (Ile) > V (Val) |
|  | [rs1363020478](https://www.ncbi.nlm.nih.gov/snp/rs1363020478) | S [TCT] > C [TGT] | NP_055306.1 | 427 | S (Ser) > C (Cys) |
|  | [rs1366122974](https://www.ncbi.nlm.nih.gov/snp/rs1366122974) | H [CAT] > R [CGT] | NP_055306.1 | 313 | H (His) > R (Arg) |
|  | [rs1366123623](https://www.ncbi.nlm.nih.gov/snp/rs1366123623) | Q [CAA] > L [CTA] | NP_055306.1 | 209 | Q (Gln) > L (Leu) |
|  | [rs1366555549](https://www.ncbi.nlm.nih.gov/snp/rs1366555549) | S [TCG] > L [TTG] | NP_055306.1 | 432 | S (Ser) > L (Leu) |
|  | [rs1371304452](https://www.ncbi.nlm.nih.gov/snp/rs1371304452) | S [AGT] > G [GGT] | NP_055306.1 | 635 | S (Ser) > G (Gly) |
|  | [rs1372909550](https://www.ncbi.nlm.nih.gov/snp/rs1372909550) | F [TTT] > L [CTT] | NP_055306.1 | 507 | F (Phe) > L (Leu) |
|  | [rs1374396490](https://www.ncbi.nlm.nih.gov/snp/rs1374396490) | S [AGC] > R [AGG] | NP_055306.1 | 355 | S (Ser) > R (Arg) |
|  | [rs1375575897](https://www.ncbi.nlm.nih.gov/snp/rs1375575897) | L [CTC] > P [CCC] | NP_055306.1 | 291 | L (Leu) > P (Pro) |
|  | [rs1378829324](https://www.ncbi.nlm.nih.gov/snp/rs1378829324) | Q [CAG] > R [CGG] | NP_055306.1 | 220 | Q (Gln) > R (Arg) |
|  | [rs1380696549](https://www.ncbi.nlm.nih.gov/snp/rs1380696549) | Y [TAC] > H [CAC] | NP_055306.1 | 483 | Y (Tyr) > H (His) |
|  | [rs1383735594](https://www.ncbi.nlm.nih.gov/snp/rs1383735594) | S [AGT] > N [AAT] | NP_055306.1 | 262 | S (Ser) > N (Asn) |
|  | [rs1383948441](https://www.ncbi.nlm.nih.gov/snp/rs1383948441) | L [CTG] > R [CGG] | NP_055306.1 | 130 | L (Leu) > R (Arg) |
|  | [rs1384054598](https://www.ncbi.nlm.nih.gov/snp/rs1384054598) | T [ACT] > S [AGT] | NP_055306.1 | 293 | T (Thr) > S (Ser) |
|  | [rs1385939134](https://www.ncbi.nlm.nih.gov/snp/rs1385939134) | K [AAG] > E [GAG] | NP_055306.1 | 674 | K (Lys) > E (Glu) |
|  | [rs1386656928](https://www.ncbi.nlm.nih.gov/snp/rs1386656928) | M [ATG] > I [ATA] | NP_055306.1 | 15 | M (Met) > I (Ile) |
|  | [rs1391902915](https://www.ncbi.nlm.nih.gov/snp/rs1391902915) | Q [CAG] > H [CAT] | NP_055306.1 | 159 | Q (Gln) > H (His) |
|  | [rs1394481073](https://www.ncbi.nlm.nih.gov/snp/rs1394481073) | I [ATC] > V [GTC] | NP_055306.1 | 671 | I (Ile) > V (Val) |
|  | [rs1394757420](https://www.ncbi.nlm.nih.gov/snp/rs1394757420) | Y [TAT] > N [AAT] | NP_055306.1 | 604 | Y (Tyr) > N (Asn) |
|  | [rs1397056325](https://www.ncbi.nlm.nih.gov/snp/rs1397056325) | D [GAC] > N [AAC] | NP_055306.1 | 701 | D (Asp) > N (Asn) |
|  | [rs1400180568](https://www.ncbi.nlm.nih.gov/snp/rs1400180568) | T [ACC] > A [GCC] | NP_055306.1 | 593 | T (Thr) > A (Ala) |
|  | [rs1402486267](https://www.ncbi.nlm.nih.gov/snp/rs1402486267) | Q [CAA] > E [GAA] | NP_055306.1 | 225 | Q (Gln) > E (Glu) |
|  | [rs1403341767](https://www.ncbi.nlm.nih.gov/snp/rs1403341767) | L [CTT] > F [TTT] | NP_055306.1 | 121 | L (Leu) > F (Phe) |
|  | [rs1406288053](https://www.ncbi.nlm.nih.gov/snp/rs1406288053) | N [AAT] > Y [TAT] | NP_055306.1 | 632 | N (Asn) > Y (Tyr) |
|  | [rs1407370674](https://www.ncbi.nlm.nih.gov/snp/rs1407370674) | Q [CAG] > R [CGG] | NP_055306.1 | 159 | Q (Gln) > R (Arg) |
|  | [rs1416091793](https://www.ncbi.nlm.nih.gov/snp/rs1416091793) | S [AGT] > N [AAT] | NP_055306.1 | 79 | S (Ser) > N (Asn |
|  | [rs1419544157](https://www.ncbi.nlm.nih.gov/snp/rs1419544157) | P [CCA] > S [TCA] | NP_055306.1 | 84 | P (Pro) > S (Ser) |
|  | [rs1423083583](https://www.ncbi.nlm.nih.gov/snp/rs1423083583) | D [GAC] > Y [TAC] | NP_055306.1 | 39 | D (Asp) > Y (Tyr) |
|  | [rs1425319727](https://www.ncbi.nlm.nih.gov/snp/rs1425319727) | S [TCC] > F [TTC] | NP_055306.1 | 315 | S (Ser) > F (Phe) |
|  | [rs1428334171](https://www.ncbi.nlm.nih.gov/snp/rs1428334171) | V [GTC] > F [TTC] | NP_055306.1 | 112 | V (Val) > F (Phe) |
|  | [rs1433376279](https://www.ncbi.nlm.nih.gov/snp/rs1433376279) | E [GAG] > K [AAG] | NP_055306.1 | 415 | E (Glu) > K (Lys) |
|  | [rs1436939063](https://www.ncbi.nlm.nih.gov/snp/rs1436939063) | M [ATG] > K [AAG] | NP_055306.1 | 94 | M (Met) > K (Lys) |
|  | [rs1438024604](https://www.ncbi.nlm.nih.gov/snp/rs1438024604) | E [GAG] > V [GTG] | NP_055306.1 | 265 | E (Glu) > V (Val) |
|  | [rs1438512681](https://www.ncbi.nlm.nih.gov/snp/rs1438512681) | P [CCA] > L [CTA] | NP_055306.1 | 245 | P (Pro) > L (Leu) |
|  | [rs1441263206](https://www.ncbi.nlm.nih.gov/snp/rs1441263206) | H [CAT] > R [CGT] | NP_055306.1 | 147 | H (His) > R (Arg) |
|  | [rs1442230907](https://www.ncbi.nlm.nih.gov/snp/rs1442230907) | A [GCA] > V [GTA] | NP_055306.1 | 680 | A (Ala) > V (Val) |
|  | [rs1445779721](https://www.ncbi.nlm.nih.gov/snp/rs1445779721) | P [CCC] > H [CAC] | NP_055306.1 | 486 | P (Pro) > H (His) |
|  | [rs1447075960](https://www.ncbi.nlm.nih.gov/snp/rs1447075960) | V [GTA] > I [ATA] | NP_055306.1 | 47 | V (Val) > I (Ile) |
|  | [rs1447805795](https://www.ncbi.nlm.nih.gov/snp/rs1447805795) | E [GAG] > K [AAG] | NP_055306.1 | 334 | E (Glu) > K (Lys) |
|  | [rs1448096881](https://www.ncbi.nlm.nih.gov/snp/rs1448096881) | K [AAA] > R [AGA] | NP_055306.1 | 74 | K (Lys) > R (Arg) |
|  | [rs1452002022](https://www.ncbi.nlm.nih.gov/snp/rs1452002022) | S [AGT] > G [GGT] | NP_055306.1 | 696 | S (Ser) > G (Gly) |
|  | [rs1452181228](https://www.ncbi.nlm.nih.gov/snp/rs1452181228) | A [GCA] > T [ACA] | NP_055306.1 | 61 | A (Ala) > T (Thr) |
|  | [rs1458512225](https://www.ncbi.nlm.nih.gov/snp/rs1458512225) | I [ATA] > T [ACA] | NP_055306.1 | 475 | I (Ile) > T (Thr) |
|  | [rs1459605752](https://www.ncbi.nlm.nih.gov/snp/rs1459605752) | H [CAC] > R [CGC] | NP_055306.1 | 651 | H (His) > R (Arg) |
|  | [rs1461838608](https://www.ncbi.nlm.nih.gov/snp/rs1461838608) | D [GAT] > E [GAA] | NP_055306.1 | 650 | D (Asp) > E (Glu) |
|  | [rs1463421426](https://www.ncbi.nlm.nih.gov/snp/rs1463421426) | A [GCA] > V [GTA] | NP_055306.1 | 545 | A (Ala) > V (Val) |
|  | [rs1464232313](https://www.ncbi.nlm.nih.gov/snp/rs1464232313) | S [TCG] > W [TGG] | NP_055306.1 | 331 | S (Ser) > W (Trp) |
|  | [rs1468750371](https://www.ncbi.nlm.nih.gov/snp/rs1468750371) | T [ACA] > K [AAA] | NP_055306.1 | 691 | T (Thr) > K (Lys) |
|  | [rs1476893897](https://www.ncbi.nlm.nih.gov/snp/rs1476893897) | K [AAG] > M [ATG] | NP_055306.1 | 365 | K (Lys) > M (Met) |
|  | [rs1476956484](https://www.ncbi.nlm.nih.gov/snp/rs1476956484) | T [ACA] > A [GCA] | NP_055306.1 | 9 | T (Thr) > A (Ala) |
|  | [rs1480440632](https://www.ncbi.nlm.nih.gov/snp/rs1480440632) | A [GCC] > S [TCC] | NP_055306.1 | 120 | A (Ala) > S (Ser) |
|  | [rs1483420106](https://www.ncbi.nlm.nih.gov/snp/rs1483420106) | D [GAC] > E [GAG] | NP_055306.1 | 329 | D (Asp) > E (Glu) |
|  | [rs1563044670](https://www.ncbi.nlm.nih.gov/snp/rs1563044670) | Q [CAG] > H [CAC] | NP_055306.1 | 160 | Q (Gln) > H (His) |
|  | [rs1563046071](https://www.ncbi.nlm.nih.gov/snp/rs1563046071) | Q [CAA] > P [CCA] | NP_055306.1 | 196 | Q (Gln) > P (Pro) |
|  | [rs1563054003](https://www.ncbi.nlm.nih.gov/snp/rs1563054003) | K [AAA] > E [GAA] | NP_055306.1 | 306 | K (Lys) > E (Glu) |
|  | [rs1563061750](https://www.ncbi.nlm.nih.gov/snp/rs1563061750) | P [CCT] > R [CGT] | NP_055306.1 | 422 | P (Pro) > R (Arg) |
|  | [rs1563064369](https://www.ncbi.nlm.nih.gov/snp/rs1563064369) | N [AAT] > S [AGT] | NP_055306.1 | 424 | N (Asn) > S (Ser) |
|  | [rs1563064499](https://www.ncbi.nlm.nih.gov/snp/rs1563064499) | T [ACC] > A [GCC] | NP_055306.1 | 448 | T (Thr) > A (Ala) |
|  | [rs1584971788](https://www.ncbi.nlm.nih.gov/snp/rs1584971788) | L [CTT] > R [CGT] | NP_055306.1 | 251 | L (Leu) > R (Arg) |
|  | [rs1584983348](https://www.ncbi.nlm.nih.gov/snp/rs1584983348) | T [ACT] > A [GCT] | NP_055306.1 | 293 | T (Thr) > A (Ala) |
|  | [rs1584983574](https://www.ncbi.nlm.nih.gov/snp/rs1584983574) | L [CTA] > P [CCA] | NP_055306.1 | 324 | L (Leu) > P (Pro) |
|  | [rs1585001758](https://www.ncbi.nlm.nih.gov/snp/rs1585001758) | T [ACC] > P [CCC] | NP_055306.1 | 455 | T (Thr) > P (Pro) |
|  | [rs1585001783](https://www.ncbi.nlm.nih.gov/snp/rs1585001783) | T [ACC] > P [CCC] | NP_055306.1 | 458 | T (Thr) > P (Pro) |
|  | [rs1585001804](https://www.ncbi.nlm.nih.gov/snp/rs1585001804) | V [GTA] > E [GAA] | NP_055306.1 | 463 | V (Val) > E (Glu) |
