# Supplementary material for: A comprehensive in silico analysis of the deleterious nonsynonymous SNPs of human FOXP2 protein
Source: PLoS One. 2022 Aug 9;17(8):e0272625. doi: 10.1371/journal.pone.0272625 (PMC9362936; doi:10.1371/journal.pone.0272625)
Supplement: S2 Table — (DOCX) [file pone.0272625.s003.docx]

**The results of five *in silico* tools that are used to analyze all of the 393 non-synonymous SNPs**

| **Serial no.** | **rsID** | **Polyphen-2** | **PROVEAN** | | **SIFT (SNPnexus)** | | **SNAP-2** | **PON P2** |  |
| --- | --- | --- | --- | --- | --- | --- | --- | --- | --- |
|  |  | **Prediction** | **PREDICTION (cutoff=-2.5)** | **SCORE** | **PREDICTION (cutoff=0.05)** | **SCORE** | **PREDICTION** | **PREDICTION** | **Probability for pathogenicity** |
|  | rs111801240 | Possibly damaging | Neutral | -0.7 | Tolerated | 0.1 | Neutral | Unknown | 0.454 |
|  | [rs121908377](https://www.ncbi.nlm.nih.gov/snp/rs121908377) | Probably damaging | Deleterious | -4.24 | Damaging | 0 | Effect | Pathogenic | 0.906 |
|  | [rs140766407](https://www.ncbi.nlm.nih.gov/snp/rs140766407) | Possibly damaging | Neutral | -2.38 | Damaging | 0 | Effect | Pathogenic | 0.938 |
|  | [rs145154396](https://www.ncbi.nlm.nih.gov/snp/rs145154396) | Possibly damaging | Neutral | -1.34 | Tolerated | 0.148 | Neutral | Unknown | 0.586 |
|  | [rs147624408](https://www.ncbi.nlm.nih.gov/snp/rs147624408) | Benign | Neutral | 0.34 | Tolerated | 0.712 | Neutral | Unknown | 0.634 |
|  | [rs182138317](https://www.ncbi.nlm.nih.gov/snp/rs182138317) | Benign | Neutral | -1.78 | Damaging | 0.01 | Effect | Unknown | 0.624 |
|  | [rs201084683](https://www.ncbi.nlm.nih.gov/snp/rs201084683) | Possibly damaging | Neutral | -2.45 | Damaging | 0.017 | Effect | Unknown | 0.578 |
|  | [rs201320940](https://www.ncbi.nlm.nih.gov/snp/rs201320940) | Possibly damaging | Neutral | -1.58 | Tolerated | 0.141 | Neutral | Unknown | 0.306 |
|  | [rs201649896](https://www.ncbi.nlm.nih.gov/snp/rs201649896) | Probably damaging | Deleterious | -3.18 | Damaging | 0.001 | Effect | Unknown | 0.557 |
|  | [rs376460299](https://www.ncbi.nlm.nih.gov/snp/rs376460299) | Possibly damaging | Deleterious | -7.25 | Damaging | 0.012 | Effect | Unknown | 0.819 |
|  | [rs529427719](https://www.ncbi.nlm.nih.gov/snp/rs529427719) | Probably damaging | Neutral | -2.16 | Damaging | 0.035 | Effect | Unknown | 0.529 |
|  | [rs564204446](https://www.ncbi.nlm.nih.gov/snp/rs564204446) | Benign | Neutral | -0.33 | Damaging | 0.025 | Neutral | neutral | 0.221 |
|  | [rs727503937](https://www.ncbi.nlm.nih.gov/snp/rs727503937) | Probably damaging | Deleterious | -3.57 | Damaging | 0.007 | Effect | Unknown | 0.745 |
|  | [rs757373725](https://www.ncbi.nlm.nih.gov/snp/rs757373725) | Benign | Neutral | -0.4 | Tolerated | 0.379 | Neutral | Unknown | 0.628 |
|  | [rs759256511](https://www.ncbi.nlm.nih.gov/snp/rs759256511) | Probably damaging | Neutral | -1.97 | Damaging | 0.025 | Effect | Unknown | 0.627 |
|  | [rs759949520](https://www.ncbi.nlm.nih.gov/snp/rs759949520) | Probably damaging | Neutral | -1.57 | Damaging | 0.004 | Effect | Unknown | 0.437 |
|  | [rs763263115](https://www.ncbi.nlm.nih.gov/snp/rs763263115) | Probably damaging | Neutral | -1.97 | Damaging | 0.002 | Effect | Unknown | 0.588 |
|  | [rs766476648](https://www.ncbi.nlm.nih.gov/snp/rs766476648) | Probably damaging | Deleterious | -2.72 | Damaging | 0.01 | Neutral | Pathogenic | 0.814 |
|  | [rs797045587](https://www.ncbi.nlm.nih.gov/snp/rs797045587) | Probably damaging | Deleterious | -2.54 | Damaging | 0 | Effect | Pathogenic | 0.854 |
|  | [rs879253772](https://www.ncbi.nlm.nih.gov/snp/rs879253772) | Probably damaging | Deleterious | -4.39 | Damaging | 0 | Effect | Pathogenic | 0.894 |
|  | [rs886061916](https://www.ncbi.nlm.nih.gov/snp/rs886061916) | Possibly damaging | Deleterious | -3.47 | Damaging | 0 | Effect | Unknown | 0.355 |
|  | [rs1563064505](https://www.ncbi.nlm.nih.gov/snp/rs1563064505) | Probably damaging | Deleterious | -4.14 | Damaging | 0.013 | Effect | Unknown | 0.64 |
|  | [rs1563067518](https://www.ncbi.nlm.nih.gov/snp/rs1563067518) | NA | Deleterious | -3.11 | NA | NA | Neutral | NA |  |
|  | [rs1584969604](https://www.ncbi.nlm.nih.gov/snp/rs1584969604) | Possibly damaging | Neutral | -0.4 | Damaging | 0.022 | Neutral | Unknown | 0.517 |
|  | [rs74507296](https://www.ncbi.nlm.nih.gov/snp/rs74507296) | Probably damaging | Deleterious | -2.87 | Damaging | 0.001 | Neutral | Unknown | 0.582 |
|  | [rs112732214](https://www.ncbi.nlm.nih.gov/snp/rs112732214) | Probably damaging | Deleterious | -5.52 | Damaging | 0 | Effect | Pathogenic | 0.915 |
|  | [rs138374374](https://www.ncbi.nlm.nih.gov/snp/rs138374374) | Benign | Neutral | -0.91 | Tolerated | 0.4 | Neutral | Unknown | 0.464 |
|  | [rs141431326](https://www.ncbi.nlm.nih.gov/snp/rs141431326) | Probably damaging | Neutral | -2.46 | Damaging | 0.047 | Neutral | Unknown | 0.782 |
|  | [rs142199218](https://www.ncbi.nlm.nih.gov/snp/rs142199218) | Benign | Neutral | 0.39 | Tolerated | 0.083 | Neutral | Unknown | 0.255 |
|  | rs143019903 | Possibly damaging | Deleterious | -2.7 | Tolerated | 0.06 | Neutral | neutral | 0.147 |
|  | rs145254341 | Benign | Neutral | -0.4 | Damaging | 0.004 | Neutral | Unknown | 0.607 |
|  | rs148201242 | Possibly damaging | Neutral | -1.4 | Damaging | 0.002 | Effect | neutral | 0.205 |
|  | rs149805525 | Probably damaging | Neutral | -2.44 | Damaging | 0.017 | Effect | Unknown | 0.653 |
|  | rs150907165 | Probably damaging | Deleterious | -2.56 | Damaging | 0.042 | Effect | Unknown | 0.639 |
|  | rs181670107 | Probably damaging | Neutral | -0.99 | Damaging | 0.036 | Effect | Unknown | 0.404 |
|  | rs185960561 | Probably damaging | Neutral | -1.86 | Damaging | 0.001 | Effect | Pathogenic | 0.817 |
|  | rs189863975 | Probably damaging | Neutral | -1.96 | Tolerated | 0.054 | Effect | Unknown | 0.56 |
|  | rs199776572 | Probably damaging | Deleterious | -2.99 | Damaging | 0.004 | Effect | Pathogenic | 0.889 |
|  | [rs202008325](https://www.ncbi.nlm.nih.gov/snp/rs202008325) | Benign | Neutral | -0.69 | Tolerated | 0.269 | Neutral | Unknown | 0.64 |
|  | rs368203434 | Possibly damaging | Deleterious | -2.5 | Tolerated | 0.054 | Effect | Unknown | 0.378 |
|  | rs369313543 | Probably damaging | Deleterious | -6.08 | Damaging | 0.045 | Neutral | Pathogenic | 0.936 |
|  | rs369839969 | Benign | Neutral | -1.8 | Tolerated | 0.062 | Neutral | Unknown | 0.709 |
|  | rs372477540 | Possibly damaging | Neutral | -1.62 | Tolerated | 0.074 | Effect | Unknown | 0.566 |
|  | rs372704196 | Benign | Deleterious | -4.95 | Damaging | 0.003 | Neutral | Pathogenic | 0.878 |
|  | rs375163729 | Possibly damaging | Neutral | -1 | Tolerated | 0.364 | Neutral | Unknown | 0.306 |
|  | rs377217556 | Benign | Neutral | -0.35 | Tolerated | 0.422 | Neutral | Unknown | 0.649 |
|  | rs377372067 | Probably damaging | Neutral | -2.04 | Damaging | 0.001 | Effect | Unknown | 0.513 |
|  | rs377420314 | Probably damaging | Deleterious | -5.23 | Damaging | 0 | Effect | Pathogenic | 0.922 |
|  | rs377588856 | Possibly damaging | Neutral | -0.95 | Damaging | 0.005 | Neutral | Unknown | 0.505 |
|  | rs531504911 | Possibly damaging | Neutral | -0.59 | Tolerated | 0.4 | Neutral | Unknown | 0.73 |
|  | rs541820585 | Benign | Deleterious | -2.7 | Damaging | 0.012 | Effect | Unknown | 0.684 |
|  | rs555128980 | Benign | Neutral | -2.26 | Damaging | 0.015 | Neutral | Unknown | 0.255 |
|  | rs562313396 | Probably damaging | Deleterious | -5.91 | Damaging | 0.014 | Effect | Unknown | 0.577 |
|  | rs563897082 | Benign | Neutral | -0.34 | Tolerated | 0.193 | Neutral | neutral | 0.178 |
|  | rs565909014 | Benign | Neutral | 0.4 | Tolerated | 0.953 | Neutral | Unknown | 0.551 |
|  | rs566961630 | Probably damaging | Deleterious | -6.69 | Damaging | 0 | Effect | Pathogenic | 0.92 |
|  | rs576347421 | Possibly damaging | Neutral | -2.08 | Damaging | 0.002 | Effect | Pathogenic | 0.796 |
|  | rs576347421 | Possibly damaging | Neutral | -1.35 | Damaging | 0.003 | Effect | Pathogenic | 0.764 |
|  | rs745342916 | Probably damaging | Deleterious | -6.39 | Damaging | 0 | Effect | Pathogenic | 0.871 |
|  | [rs745618497](https://www.ncbi.nlm.nih.gov/snp/rs745618497) | Probably damaging | Neutral | -1.03 | Damaging | 0.008 | Effect | Pathogenic | 0.872 |
|  | [rs745987986](https://www.ncbi.nlm.nih.gov/snp/rs745987986) | Benign | Neutral | -0.13 | Tolerated | 0.699 | Neutral | Unknown | 0.3 |
|  | [rs746400429](https://www.ncbi.nlm.nih.gov/snp/rs746400429) | Benign | Neutral | -1.07 | Damaging | 0.003 | Effect | Pathogenic | 0.872 |
|  | [rs746524111](https://www.ncbi.nlm.nih.gov/snp/rs746524111) | Possibly damaging | Neutral | -0.89 | Damaging | 0.013 | Effect | Unknown | 0.462 |
|  | [rs746703454](https://www.ncbi.nlm.nih.gov/snp/rs746703454) | Probably damaging | Neutral | -2.49 | Damaging | 0 | Effect | Unknown | 0.792 |
|  | [rs746884663](https://www.ncbi.nlm.nih.gov/snp/rs746884663) | Benign | Neutral | 0.06 | Tolerated | 0.767 | Neutral | Unknown | 0.17 |
|  | [rs747155760](https://www.ncbi.nlm.nih.gov/snp/rs747155760) | Benign | Neutral | -0.82 | Tolerated | 0.26 | Neutral | Unknown | 0.309 |
|  | [rs747467925](https://www.ncbi.nlm.nih.gov/snp/rs747467925) | Probably damaging | Neutral | -0.38 | Damaging | 0.049 | Neutral | Unknown | 0.597 |
|  | [rs748073993](https://www.ncbi.nlm.nih.gov/snp/rs748073993) | Probably damaging | Neutral | -1.75 | Damaging | 0.002 | Neutral | Unknown | 0.647 |
|  | [rs748231233](https://www.ncbi.nlm.nih.gov/snp/rs748231233) | Possibly damaging | Neutral | -1.67 | Tolerated | 0.053 | Neutral | Unknown | 0.67 |
|  | [rs748314629](https://www.ncbi.nlm.nih.gov/snp/rs748314629) | Possibly damaging | Neutral | -1.72 | Damaging | 0.014 | Neutral | Unknown | 0.645 |
|  | [rs748597582](https://www.ncbi.nlm.nih.gov/snp/rs748597582) | Possibly damaging | Deleterious | -2.61 | Tolerated | 0.313 | Effect | Unknown | 0.682 |
|  | [rs748878832](https://www.ncbi.nlm.nih.gov/snp/rs748878832) | Probably damaging | Deleterious | -6.36 | Damaging | 0 | Effect | Unknown | 0.656 |
|  | [rs749254802](https://www.ncbi.nlm.nih.gov/snp/rs749254802) | Possibly damaging | Deleterious | -3.26 | Damaging | 0.004 | Neutral | Unknown | 0.256 |
|  | [rs749278394](https://www.ncbi.nlm.nih.gov/snp/rs749278394) | Possibly damaging | Deleterious | -5.9 | Damaging | 0.022 | Effect | Unknown | 0.76 |
|  | [rs749932113](https://www.ncbi.nlm.nih.gov/snp/rs749932113) | Probably damaging | Neutral | -1 | Damaging | 0.003 | Neutral | Unknown | 0.258 |
|  | [rs750028450](https://www.ncbi.nlm.nih.gov/snp/rs750028450) | Probably damaging | Neutral | -2.22 | Damaging | 0.003 | Neutral | Pathogenic | 0.884 |
|  | [rs750287608](https://www.ncbi.nlm.nih.gov/snp/rs750287608) | Probably damaging | Neutral | -2.22 | Damaging | 0.006 | Effect | Pathogenic | 0.934 |
|  | [rs750505654](https://www.ncbi.nlm.nih.gov/snp/rs750505654) | Probably damaging | Neutral | -0.48 | Tolerated | 0.457 | Neutral | Pathogenic | 0.801 |
|  | [rs750582959](https://www.ncbi.nlm.nih.gov/snp/rs750582959) | Benign | Neutral | -0.56 | Tolerated | 0.394 | Neutral | Neutral | 0.132 |
|  | [rs750701057](https://www.ncbi.nlm.nih.gov/snp/rs750701057) | Benign | Neutral | -1.46 | Tolerated | 0.082 | Neutral | Unknown | 0.309 |
|  | [rs750736305](https://www.ncbi.nlm.nih.gov/snp/rs750736305) | Benign | Neutral | -0.39 | Tolerated | 0.383 | Neutral | Unknown | 0.698 |
|  | [rs751166815](https://www.ncbi.nlm.nih.gov/snp/rs751166815) | Benign | Neutral | -1.79 | Damaging | 0.02 | Neutral | Unknown | 0.277 |
|  | [rs751732210](https://www.ncbi.nlm.nih.gov/snp/rs751732210) | Probably damaging | Deleterious | -2.53 | Damaging | 0.01 | Effect | Unknown | 0.587 |
|  | [rs751931499](https://www.ncbi.nlm.nih.gov/snp/rs751931499) | Probably damaging | Deleterious | -6.21 | Damaging | 0.001 | Effect | Pathogenic | 0.94 |
|  | [rs752426529](https://www.ncbi.nlm.nih.gov/snp/rs752426529) | Benign | Neutral | -0.9 | Tolerated | 0.139 | Neutral | Unknown | 0.505 |
|  | [rs752528443](https://www.ncbi.nlm.nih.gov/snp/rs752528443) | Probably damaging | Neutral | -1.04 | Tolerated | 0.079 | Effect | Unknown | 0.594 |
|  | [rs753356750](https://www.ncbi.nlm.nih.gov/snp/rs753356750) | Possibly damaging | Neutral | -1.27 | Damaging | 0.006 | Neutral | Unknown | 0.427 |
|  | [rs753394697](https://www.ncbi.nlm.nih.gov/snp/rs753394697) | Benign | Neutral | -1.02 | Damaging | 0.021 | Neutral | Neutral | 0.198 |
|  | [rs753640569](https://www.ncbi.nlm.nih.gov/snp/rs753640569) | Benign | Neutral | -1.45 | Tolerated | 0.107 | Neutral | Unknown | 0.349 |
|  | [rs753643915](https://www.ncbi.nlm.nih.gov/snp/rs753643915) | Benign | Neutral | -1.9 | Tolerated | 0.063 | Neutral | Unknown | 0.456 |
|  | [rs753658194](https://www.ncbi.nlm.nih.gov/snp/rs753658194) | Benign | Deleterious | -3.35 | Tolerated | 0.198 | Neutral | Pathogenic | 0.95 |
|  | [rs753732678](https://www.ncbi.nlm.nih.gov/snp/rs753732678) | Possibly damaging | Neutral | -1.17 | Tolerated | 0.055 | Neutral | Pathogenic | 0.94 |
|  | [rs753857220](https://www.ncbi.nlm.nih.gov/snp/rs753857220) | Possibly damaging | Neutral | -1.59 | Damaging | 0.013 | Neutral | Pathogenic | 0.873 |
|  | [rs754271344](https://www.ncbi.nlm.nih.gov/snp/rs754271344) | Probably damaging | Neutral | -1.54 | Damaging | 0.021 | Neutral | Unknown | 0.43 |
|  | [rs754361203](https://www.ncbi.nlm.nih.gov/snp/rs754361203) | Possibly damaging | Deleterious | -2.95 | Damaging | 0.015 | Effect | Unknown | 0.313 |
|  | [rs754927033](https://www.ncbi.nlm.nih.gov/snp/rs754927033) | Benign | Deleterious | -2.72 | Damaging | 0.047 | Effect | Unknown | 0.792 |
|  | [rs755297474](https://www.ncbi.nlm.nih.gov/snp/rs755297474) | Probably damaging | Deleterious | -8.55 | Damaging | 0 | Effect | Pathogenic | 0.922 |
|  | [rs755668650](https://www.ncbi.nlm.nih.gov/snp/rs755668650) | Benign | Neutral | -2.46 | Damaging | 0.001 | Neutral | Unknown | 0.442 |
|  | [rs755825043](https://www.ncbi.nlm.nih.gov/snp/rs755825043) | Possibly damaging | Deleterious | -3.22 | Damaging | 0.003 | Effect | Unknown | 0.618 |
|  | [rs755829229](https://www.ncbi.nlm.nih.gov/snp/rs755829229) | Benign | Neutral | -1.96 | Tolerated | 0.223 | Neutral | Unknown | 0.035 |
|  | [rs755981069](https://www.ncbi.nlm.nih.gov/snp/rs755981069) | Probably damaging | Deleterious | -2.62 | Damaging | 0 | Effect | Unknown | 0.325 |
|  | [rs756019068](https://www.ncbi.nlm.nih.gov/snp/rs756019068) | Probably damaging | Neutral | -2.32 | Tolerated | 0.082 | Effect | Pathogenic | 0.831 |
|  | [rs756184095](https://www.ncbi.nlm.nih.gov/snp/rs756184095) | Probably damaging | Deleterious | -3.56 | Damaging | 0 | Effect | Unknown | 0.557 |
|  | [rs756555227](https://www.ncbi.nlm.nih.gov/snp/rs756555227) | Possibly damaging | Neutral | -0.89 | Damaging | 0.005 | Neutral | Unknown | 0.594 |
|  | [rs757255710](https://www.ncbi.nlm.nih.gov/snp/rs757255710) | Possibly damaging | Neutral | -0.96 | Tolerated | 0.561 | Neutral | Unknown | 0.486 |
|  | [rs757270050](https://www.ncbi.nlm.nih.gov/snp/rs757270050) | Probably damaging | Deleterious | -5.21 | Damaging | 0.001 | Effect | Unknown | 0.729 |
|  | [rs757351650](https://www.ncbi.nlm.nih.gov/snp/rs757351650) | Probably damaging | Neutral | -1.84 | Damaging | 0.027 | Effect | Unknown | 0.436 |
|  | [rs758052155](https://www.ncbi.nlm.nih.gov/snp/rs758052155) | Benign | Neutral | -0.93 | Tolerated | 0.123 | Effect | Unknown | 0.48 |
|  | [rs758080021](https://www.ncbi.nlm.nih.gov/snp/rs758080021) | Benign | Deleterious | -3.28 | Damaging | 0.044 | Effect | Unknown | 0.555 |
|  | [rs758423327](https://www.ncbi.nlm.nih.gov/snp/rs758423327) | Possibly damaging | Neutral | 0.62 | Tolerated | 0.29 | Neutral | Neutral | 0.217 |
|  | [rs758427088](https://www.ncbi.nlm.nih.gov/snp/rs758427088) | Probably damaging | Deleterious | -3.08 | Damaging | 0.002 | Effect | Pathogenic | 0.962 |
|  | [rs758513311](https://www.ncbi.nlm.nih.gov/snp/rs758513311) | Probably damaging | Deleterious | -8.7 | Damaging | 0.001 | Effect | Pathogenic | 0.94 |
|  | [rs758714987](https://www.ncbi.nlm.nih.gov/snp/rs758714987) | Benign | Neutral | -0.17 | Tolerated | 0.065 | Neutral | Unknown | 0.397 |
|  | [rs760114813](https://www.ncbi.nlm.nih.gov/snp/rs760114813) | Benign | Neutral | -1.36 | Tolerated | 0.066 | Neutral |  |  |
|  | [rs760226272](https://www.ncbi.nlm.nih.gov/snp/rs760226272) | Benign | Neutral | -0.26 | Tolerated | 0.062 | Neutral | Neutral | 0.173 |
|  | [rs760596736](https://www.ncbi.nlm.nih.gov/snp/rs760596736) | Possibly damaging | Deleterious | -8.14 | Damaging | 0.001 | Effect | Pathogenic | 0.913 |
|  | [rs760842249](https://www.ncbi.nlm.nih.gov/snp/rs760842249) | Probably damaging | Neutral | -2.09 | Damaging | 0.001 | Effect | Unknown | 0.592 |
|  | [rs761170451](https://www.ncbi.nlm.nih.gov/snp/rs761170451) | Benign | Neutral | -2.42 | Tolerated | 0.065 | Effect | Unknown | 0.59 |
|  | [rs761174159](https://www.ncbi.nlm.nih.gov/snp/rs761174159) | Benign | Deleterious | -3.96 | Damaging | 0.046 | Effect | Unknown | 0.571 |
|  | [rs761262072](https://www.ncbi.nlm.nih.gov/snp/rs761262072) | Possibly damaging | Neutral | -1.9 | Damaging | 0.03 | Effect | Unknown | 0.648 |
|  | [rs762007110](https://www.ncbi.nlm.nih.gov/snp/rs762007110) | Probably damaging | Neutral | -0.74 | Damaging | 0.014 | Neutral | Unknown | 0.383 |
|  | [rs762414429](https://www.ncbi.nlm.nih.gov/snp/rs762414429) | Benign | Neutral | -1.05 | Tolerated | 0.36 | Effect | Unknown | 0.817 |
|  | [rs762564041](https://www.ncbi.nlm.nih.gov/snp/rs762564041) | Probably damaging | Deleterious | -7.35 | Damaging | 0 | Effect | Pathogenic | 0.873 |
|  | [rs762699659](https://www.ncbi.nlm.nih.gov/snp/rs762699659) | Probably damaging | Neutral | -2.42 | Damaging | 0 | Effect | Pathogenic | 0.92 |
|  | [rs762817913](https://www.ncbi.nlm.nih.gov/snp/rs762817913) | Probably damaging | Neutral | -2.03 | Damaging | 0.002 | Effect | neutral | 0.172 |
|  | [rs763246182](https://www.ncbi.nlm.nih.gov/snp/rs763246182) | Possibly damaging | Deleterious | -3.58 | Damaging | 0 | Effect | Unknown | 0.601 |
|  | [rs763302537](https://www.ncbi.nlm.nih.gov/snp/rs763302537) | Benign | Neutral | -0.7 | Tolerated | 0.142 | Neutral | Unknown | 0.683 |
|  | [rs763449275](https://www.ncbi.nlm.nih.gov/snp/rs763449275) | Probably damaging | Deleterious | -3.32 | Damaging | 0 | Effect | Unknown | 0.342 |
|  | [rs763457983](https://www.ncbi.nlm.nih.gov/snp/rs763457983) | Benign | Neutral | -0.73 | Damaging | 0.025 | Effect | Pathogenic | 0.872 |
|  | [rs763537390](https://www.ncbi.nlm.nih.gov/snp/rs763537390) | Benign | Neutral | -1.19 | Tolerated | 0.275 | Neutral | Pathogenic | 0.872 |
|  | [rs763629604](https://www.ncbi.nlm.nih.gov/snp/rs763629604) | Benign | Neutral | -0.61 | Damaging | 0.018 | Neutral | Unknown | 0.339 |
|  | [rs763694805](https://www.ncbi.nlm.nih.gov/snp/rs763694805) | Benign | Neutral | -0.45 | Damaging | 0.042 | Neutral | Unknown | 0.462 |
|  | [rs763694805](https://www.ncbi.nlm.nih.gov/snp/rs763694805) | Benign | Neutral | 0.42 | Tolerated | 0.708 | Neutral | Neutral | 0.15 |
|  | [rs764109995](https://www.ncbi.nlm.nih.gov/snp/rs764109995) | Possibly damaging | Neutral | -1.13 | Damaging | 0.041 | Neutral | Unknown | 0.55 |
|  | [rs764574245](https://www.ncbi.nlm.nih.gov/snp/rs764574245) | Benign | Neutral | -0.21 | Tolerated | 0.617 | Neutral | Neutral | 0.149 |
|  | [rs764574245](https://www.ncbi.nlm.nih.gov/snp/rs764574245) | Probably damaging | Neutral | -2.37 | Damaging | 0.007 | Effect | Unknown | 0.43 |
|  | [rs765157455](https://www.ncbi.nlm.nih.gov/snp/rs765157455) | Probably damaging | Deleterious | -5.47 | Damaging | 0.001 | Effect | Pathogenic | 0.841 |
|  | [rs765157455](https://www.ncbi.nlm.nih.gov/snp/rs765157455) | Probably damaging | Deleterious | -6.78 | Damaging | 0 | Effect | Pathogenic | 0.935 |
|  | [rs765574065](https://www.ncbi.nlm.nih.gov/snp/rs765574065) | Possibly damaging | Deleterious | -2.8 | Damaging | 0.002 | Effect | Unknown | 0.653 |
|  | [rs765647678](https://www.ncbi.nlm.nih.gov/snp/rs765647678) | Probably damaging | Neutral | -1.55 | Damaging | 0.003 | Effect | Unknown | 0.773 |
|  | [rs765980369](https://www.ncbi.nlm.nih.gov/snp/rs765980369) | Probably damaging | Deleterious | -2.82 | Damaging | 0.02 | Neutral | Unknown | 0.344 |
|  | [rs765980960](https://www.ncbi.nlm.nih.gov/snp/rs765980960) | Possibly damaging | Neutral | -1.09 | Tolerated | 0.052 | Effect | Pathogenic | 0.844 |
|  | [rs766020871](https://www.ncbi.nlm.nih.gov/snp/rs766020871) | Probably damaging | Neutral | -2.18 | Tolerated | 0.055 | Neutral | Unknown | 0.757 |
|  | [rs766803095](https://www.ncbi.nlm.nih.gov/snp/rs766803095) | Probably damaging | Neutral | -0.96 | Tolerated | 0.146 | Neutral | Unknown | 0.701 |
|  | [rs766956626](https://www.ncbi.nlm.nih.gov/snp/rs766956626) | Benign | Neutral | -0.75 | Tolerated | 0.209 | Neutral | Neutral | 0.247 |
|  | [rs766965457](https://www.ncbi.nlm.nih.gov/snp/rs766965457) | Benign | Deleterious | -3.3 | Damaging | 0.012 | Effect | Unknown | 0.264 |
|  | [rs767291302](https://www.ncbi.nlm.nih.gov/snp/rs767291302) | Benign | Neutral | -0.94 | Tolerated | 0.143 | Neutral | Unknown | 0.415 |
|  | [rs767291302](https://www.ncbi.nlm.nih.gov/snp/rs767291302) | Benign | Neutral | 0.31 | Tolerated | 0.56 | Neutral | Unknown | 0.324 |
|  | [rs767652094](https://www.ncbi.nlm.nih.gov/snp/rs767652094) | Probably damaging | Neutral | -2.03 | Tolerated | 0.197 | Neutral | Unknown | 0.332 |
|  | [rs767652094](https://www.ncbi.nlm.nih.gov/snp/rs767652094) | Probably damaging | Deleterious | -2.97 | Damaging | 0.001 | Effect | Unknown | 0.362 |
|  | [rs768186362](https://www.ncbi.nlm.nih.gov/snp/rs768186362) | Benign | Neutral | -1.58 | Damaging | 0.035 | Effect | Unknown | 0.624 |
|  | [rs768186362](https://www.ncbi.nlm.nih.gov/snp/rs768186362) | Benign | Neutral | -1.33 | Tolerated | 0.052 | Neutral | Neutral | 0.163 |
|  | [rs768728605](https://www.ncbi.nlm.nih.gov/snp/rs768728605) | Benign | Neutral | -1.56 | Damaging | 0.047 | Neutral | Neutral | 0.226 |
|  | [rs769148228](https://www.ncbi.nlm.nih.gov/snp/rs769148228) | Probably damaging | Neutral | -1.27 | Damaging | 0.005 | Effect | Pathogenic | 0.763 |
|  | [rs769218331](https://www.ncbi.nlm.nih.gov/snp/rs769218331) | Benign | Neutral | -0.58 | Tolerated | 0.27 | Neutral | unknown | 0.267 |
|  | [rs769296657](https://www.ncbi.nlm.nih.gov/snp/rs769296657) | Possibly damaging | Deleterious | -4.55 | Tolerated | 0.067 | Effect | Pathogenic | 0.892 |
|  | [rs769641584](https://www.ncbi.nlm.nih.gov/snp/rs769641584) | Possibly damaging | Neutral | -0.72 | Damaging | 0.002 | Neutral | Unknown | 0.594 |
|  | [rs769729974](https://www.ncbi.nlm.nih.gov/snp/rs769729974) | Possibly damaging | Neutral | -1.73 | Damaging | 0.011 | Neutral | Neutral | 0.19 |
|  | [rs769729974](https://www.ncbi.nlm.nih.gov/snp/rs769729974) | Possibly damaging | Neutral | -1.5 | Tolerated | 0.053 | Neutral | Unknown | 0.229 |
|  | [rs769729974](https://www.ncbi.nlm.nih.gov/snp/rs769729974) | Benign | Neutral | -1.72 | Tolerated | 0.129 | Neutral | Neutral | 0.086 |
|  | [rs770001809](https://www.ncbi.nlm.nih.gov/snp/rs770001809) | Possibly damaging | Deleterious | -2.82 | Damaging | 0.013 | Effect | Pathogenic | 0.839 |
|  | [rs770093841](https://www.ncbi.nlm.nih.gov/snp/rs770093841) | Benign | Neutral | -0.28 | Tolerated | 0.137 | Effect | Unknown | 0.721 |
|  | [rs770301569](https://www.ncbi.nlm.nih.gov/snp/rs770301569) | Benign | Neutral | -1.19 | Damaging | 0.011 | Effect | Pathogenic | 0.811 |
|  | [rs770756604](https://www.ncbi.nlm.nih.gov/snp/rs770756604) | Benign | Neutral | -0.94 | Damaging | 0.018 | Neutral | Unknown | 0.625 |
|  | [rs770923990](https://www.ncbi.nlm.nih.gov/snp/rs770923990) | Benign | Neutral | 0.09 | Tolerated | 0.106 | Neutral | Neutral | 0.185 |
|  | [rs771268489](https://www.ncbi.nlm.nih.gov/snp/rs771268489) | Benign | Neutral | -0.05 | Tolerated | 0.631 | Neutral | Unknown | 0.425 |
|  | [rs771314598](https://www.ncbi.nlm.nih.gov/snp/rs771314598) | Benign | Neutral | -2.02 | Tolerated | 0.206 | Neutral | Unknown | 0.42 |
|  | [rs771438075](https://www.ncbi.nlm.nih.gov/snp/rs771438075) | Benign | Deleterious | -2.94 | Damaging | 0.003 | Neutral | Unknown | 0.445 |
|  | [rs771840840](https://www.ncbi.nlm.nih.gov/snp/rs771840840) | Benign | Neutral | -0.65 | Tolerated | 0.496 | Neutral | Unknown | 0.252 |
|  | [rs772009456](https://www.ncbi.nlm.nih.gov/snp/rs772009456) | Benign | Neutral | -0.01 | Tolerated | 0.492 | Neutral | Unknown | 0.403 |
|  | [rs772294351](https://www.ncbi.nlm.nih.gov/snp/rs772294351) | Probably damaging | Neutral | -1.03 | Damaging | 0.001 | Effect | Unknown | 0.63 |
|  | [rs772694863](https://www.ncbi.nlm.nih.gov/snp/rs772694863) | Probably damaging | Deleterious | -5.95 | Damaging | 0.001 | Effect | Pathogenic | 0.947 |
|  | [rs772881522](https://www.ncbi.nlm.nih.gov/snp/rs772881522) | Possibly damaging | Deleterious | -2.95 | Damaging | 0.003 | Effect | Unknown | 0.413 |
|  | [rs773700650](https://www.ncbi.nlm.nih.gov/snp/rs773700650) | Benign | Neutral | -2.4 | Damaging | 0.01 | Neutral | Unknown | 0.486 |
|  | [rs773759127](https://www.ncbi.nlm.nih.gov/snp/rs773759127) | Probably damaging | Deleterious | -4.26 | Damaging | 0.001 | Effect | Unknown | 0.782 |
|  | [rs773874222](https://www.ncbi.nlm.nih.gov/snp/rs773874222) | Probably damaging | Deleterious | -5.47 | Damaging | 0.001 | Effect | Unknown | 0.728 |
|  | [rs773947565](https://www.ncbi.nlm.nih.gov/snp/rs773947565) | Possibly damaging | Deleterious | -2.5 | Tolerated | 0.079 | Effect | Pathogenic | 0.9 |
|  | [rs774292922](https://www.ncbi.nlm.nih.gov/snp/rs774292922) | Possibly damaging | Deleterious | -2.59 | Tolerated | 0.092 | Neutral | Unknown | 0.766 |
|  | [rs774304280](https://www.ncbi.nlm.nih.gov/snp/rs774304280) | Benign | Neutral | -2.32 | Tolerated | 0.238 | Neutral | Neutral | 0.159 |
|  | [rs774408585](https://www.ncbi.nlm.nih.gov/snp/rs774408585) | Benign | Neutral | 0.29 | Tolerated | 0.584 | Neutral | Neutral | 0.23 |
|  | [rs774842735](https://www.ncbi.nlm.nih.gov/snp/rs774842735) | Benign | Neutral | 0.05 | Damaging | 0.041 | Neutral | Unknown | 0.72 |
|  | [rs774842735](https://www.ncbi.nlm.nih.gov/snp/rs774842735) | Benign | Neutral | 0.38 | Tolerated | 0.807 | Neutral | Unknown | 0.458 |
|  | [rs775655580](https://www.ncbi.nlm.nih.gov/snp/rs775655580) | Possibly damaging | Neutral | -2.22 | Damaging | 0.009 | Effect | Pathogenic | 0.773 |
|  | [rs775730461](https://www.ncbi.nlm.nih.gov/snp/rs775730461) | Benign | Neutral | 0.18 | Tolerated | 0.497 | Neutral | Neutral | 0.158 |
|  | [rs775743904](https://www.ncbi.nlm.nih.gov/snp/rs775743904) | Benign | Neutral | -2.19 | Tolerated | 0.072 | Neutral | Pathogenic | 0.869 |
|  | [rs775819986](https://www.ncbi.nlm.nih.gov/snp/rs775819986) | Benign | Neutral | -0.09 | Tolerated | 0.791 | Neutral | Unknown | 0.569 |
|  | [rs776670223](https://www.ncbi.nlm.nih.gov/snp/rs776670223) | Possibly damaging | Neutral | 0.12 | Damaging | 0.006 | Neutral | Unknown | 0.346 |
|  | [rs776679391](https://www.ncbi.nlm.nih.gov/snp/rs776679391) | Benign | Neutral | -1.51 | Tolerated | 0.151 | Neutral | Unknown | 0.731 |
|  | [rs776679391](https://www.ncbi.nlm.nih.gov/snp/rs776679391) | Benign | Neutral | -1.44 | Tolerated | 0.335 | Neutral | Unknown | 0.817 |
|  | [rs776966569](https://www.ncbi.nlm.nih.gov/snp/rs776966569) | Benign | Neutral | 0.35 | Tolerated | 0.536 | Neutral | Unknown | 0.699 |
|  | [rs777042566](https://www.ncbi.nlm.nih.gov/snp/rs777042566) | Possibly damaging | Neutral | -0.86 | Damaging | 0.025 | Neutral | Unknown | 0.391 |
|  | [rs777132475](https://www.ncbi.nlm.nih.gov/snp/rs777132475) | Possibly damaging | Neutral | -2.44 | Tolerated | 0.051 | Neutral | Unknown | 0.387 |
|  | [rs777361904](https://www.ncbi.nlm.nih.gov/snp/rs777361904) | Benign | Neutral | -0.05 | Tolerated | 0.778 | Neutral | Unknown | 0.718 |
|  | [rs777560011](https://www.ncbi.nlm.nih.gov/snp/rs777560011) | Possibly damaging | Neutral | -2.19 | Damaging | 0.006 | Effect | Pathogenic | 0.941 |
|  | [rs778026488](https://www.ncbi.nlm.nih.gov/snp/rs778026488) | Probably damaging | Neutral | -1.15 | Tolerated | 0.084 | Neutral | Pathogenic | 0.856 |
|  | [rs778396336](https://www.ncbi.nlm.nih.gov/snp/rs778396336) | Probably damaging | Neutral | -1.69 | Tolerated | 0.14 | Neutral | Pathogenic | 0.889 |
|  | [rs778396336](https://www.ncbi.nlm.nih.gov/snp/rs778396336) | Probably damaging | Neutral | -1.66 | Tolerated | 0.06 | Neutral | Unknown | 0.651 |
|  | [rs778748061](https://www.ncbi.nlm.nih.gov/snp/rs778748061) | Probably damaging | Deleterious | -5.1 | Damaging | 0 | Neutral | Pathogenic | 0.92 |
|  | [rs778767619](https://www.ncbi.nlm.nih.gov/snp/rs778767619) | Benign | Neutral | -0.73 | Tolerated | 0.309 | Neutral | Unknown | 0.817 |
|  | [rs779157210](https://www.ncbi.nlm.nih.gov/snp/rs779157210) | Benign | Neutral | -2.17 | Tolerated | 0.06 | Effect | Unknown | 0.422 |
|  | [rs779632352](https://www.ncbi.nlm.nih.gov/snp/rs779632352) | Probably damaging | Neutral | -1.52 | Damaging | 0.002 | Effect | Pathogenic | 0.793 |
|  | [rs779754644](https://www.ncbi.nlm.nih.gov/snp/rs779754644) | Probably damaging | Deleterious | -11.12 | Damaging | 0 | Effect | Pathogenic | 0.936 |
|  | [rs779921362](https://www.ncbi.nlm.nih.gov/snp/rs779921362) | Probably damaging | Deleterious | -4.63 | Damaging | 0.009 | Effect | Pathogenic | 0.833 |
|  | [rs780212480](https://www.ncbi.nlm.nih.gov/snp/rs780212480) | Probably damaging | Deleterious | -4.13 | Damaging | 0.004 | Neutral | Pathogenic | 0.946 |
|  | [rs780371962](https://www.ncbi.nlm.nih.gov/snp/rs780371962) | Benign | Neutral | -0.64 | Tolerated | 0.087 | Neutral | Unknown | 0.364 |
|  | [rs780676420](https://www.ncbi.nlm.nih.gov/snp/rs780676420) | Benign | Neutral | -0.87 | Tolerated | 0.237 | Neutral | Neutral | 0.151 |
|  | [rs780869585](https://www.ncbi.nlm.nih.gov/snp/rs780869585) | Benign | Neutral | -0.87 | Tolerated | 0.176 | Neutral | Unknown | 0.242 |
|  | [rs781437567](https://www.ncbi.nlm.nih.gov/snp/rs781437567) | Possibly damaging | Neutral | -0.58 | Damaging | 0.024 | Neutral | Unknown | 0.222 |
|  | [rs866570059](https://www.ncbi.nlm.nih.gov/snp/rs866570059) | Possibly damaging | Neutral | -2.33 | Damaging | 0.016 | Effect | Unknown | 0.508 |
|  | [rs866894515](https://www.ncbi.nlm.nih.gov/snp/rs866894515) | Possibly damaging | Deleterious | -2.63 | Damaging | 0.001 | Effect | Unknown | 0.672 |
|  | [rs867418322](https://www.ncbi.nlm.nih.gov/snp/rs867418322) | Possibly damaging | Deleterious | -3 | Damaging | 0.004 | Effect | Unknown | 0.412 |
|  | [rs868366293](https://www.ncbi.nlm.nih.gov/snp/rs868366293) | Probably damaging | Neutral | -1.72 | Damaging | 0 | Effect | Pathogenic | 0.906 |
|  | [rs879771053](https://www.ncbi.nlm.nih.gov/snp/rs879771053) | Possibly damaging | Deleterious | -2.75 | Damaging | 0.04 | Effect | Unknown | 0.418 |
|  | [rs889178260](https://www.ncbi.nlm.nih.gov/snp/rs889178260) | Probably damaging | Neutral | -1.77 | Damaging | 0.021 | Neutral | Unknown | 0.669 |
|  | [rs889341368](https://www.ncbi.nlm.nih.gov/snp/rs889341368) | Possibly damaging | Deleterious | -2.57 | Damaging | 0.001 | Effect | Pathogenic | 0.862 |
|  | [rs895017794](https://www.ncbi.nlm.nih.gov/snp/rs895017794) | Possibly damaging | Neutral | -2.17 | Damaging | 0.041 | Effect | Unknown | 0.578 |
|  | [s899776651](https://www.ncbi.nlm.nih.gov/snp/rs899776651) | Possibly damaging | Deleterious | -2.94 | Damaging | 0.003 | Neutral | Neutral | 0.192 |
|  | [rs907456099](https://www.ncbi.nlm.nih.gov/snp/rs907456099) | Probably damaging | Neutral | -1.36 | Damaging | 0.018 | Effect | Unknown | 0.733 |
|  | [rs908446199](https://www.ncbi.nlm.nih.gov/snp/rs908446199) | Probably damaging | Deleterious | -2.83 | Damaging | 0.022 | Neutral | Pathogenic | 0.901 |
|  | [rs909392122](https://www.ncbi.nlm.nih.gov/snp/rs909392122) | Probably damaging | Deleterious | -3.51 | Damaging | 0.001 | Effect | Unknown | 0.423 |
|  | [rs915879314](https://www.ncbi.nlm.nih.gov/snp/rs915879314) | Possibly damaging | Deleterious | -2.69 | Damaging | 0.013 | Neutral | Unknown | 0.326 |
|  | [rs920208430](https://www.ncbi.nlm.nih.gov/snp/rs920208430) | Probably damaging | Neutral | -0.74 | Tolerated | 0.146 | Effect | Pathogenic | 0.935 |
|  | [rs928369081](https://www.ncbi.nlm.nih.gov/snp/rs928369081) | Probably damaging | Deleterious | -4.27 | Tolerated | 0.129 | Effect | Pathogenic | 0.847 |
|  | [rs937208031](https://www.ncbi.nlm.nih.gov/snp/rs937208031) | Possibly damaging | Neutral | -1.35 | Tolerated | 0.072 | Neutral | Pathogenic | 0.872 |
|  | [rs938443435](https://www.ncbi.nlm.nih.gov/snp/rs938443435) | Possibly damaging | Deleterious | -2.91 | Tolerated | 0.135 | Neutral | Pathogenic | 0.856 |
|  | [rs940890058](https://www.ncbi.nlm.nih.gov/snp/rs940890058) | Probably damaging | Deleterious | -2.58 | Tolerated | 0.092 | Effect | Pathogenic | 0.851 |
|  | [rs942638508](https://www.ncbi.nlm.nih.gov/snp/rs942638508) | Probably damaging | Neutral | -0.91 | Tolerated | 0.073 | Effect | Unknown | 0.496 |
|  | [rs942769559](https://www.ncbi.nlm.nih.gov/snp/rs942769559) | Possibly damaging | Neutral | -0.56 | Damaging | 0.003 | Neutral | Neutral | 0.149 |
|  | [rs944127085](https://www.ncbi.nlm.nih.gov/snp/rs944127085) | Probably damaging | Neutral | -1.96 | Tolerated | 0.324 | Neutral | Unknown | 0.564 |
|  | [rs945804979](https://www.ncbi.nlm.nih.gov/snp/rs945804979) | Benign | Neutral | -2.46 | Tolerated | 0.23 | Neutral | Unknown | 0.282 |
|  | [rs948249504](https://www.ncbi.nlm.nih.gov/snp/rs948249504) | Probably damaging | Deleterious | -3.37 | Damaging | 0 | Effect | Pathogenic | 0.906 |
|  | [rs952693445](https://www.ncbi.nlm.nih.gov/snp/rs952693445) | Benign | Neutral | -0.64 | Tolerated | 0.066 | Neutral | Unknown | 0.614 |
|  | [rs955135535](https://www.ncbi.nlm.nih.gov/snp/rs952693445) | Probably damaging | Deleterious | -6.21 | Damaging | 0.002 | Effect | Unknown | 0.658 |
|  | [rs959136398](https://www.ncbi.nlm.nih.gov/snp/rs959136398) | Benign | Neutral | -0.42 | Damaging | 0.007 | Effect | Unknown | 0.272 |
|  | [rs960171629](https://www.ncbi.nlm.nih.gov/snp/rs960171629) | Probably damaging | Neutral | -2.48 | Damaging | 0.032 | Effect | Unknown | 0.653 |
|  | [rs960747265](https://www.ncbi.nlm.nih.gov/snp/rs960747265) | Probably damaging | Neutral | -2.39 | Damaging | 0.005 | Neutral | Pathogenic | 0.919 |
|  | [rs976182523](https://www.ncbi.nlm.nih.gov/snp/rs976182523) | Benign | Deleterious | -2.89 | Damaging | 0.003 | Neutral | Neutral | 0.173 |
|  | [rs985325734](https://www.ncbi.nlm.nih.gov/snp/rs985325734) | Benign | Neutral | -2.23 | Damaging | 0.037 | Neutral | Pathogenic | 0.94 |
|  | [rs1003245699](https://www.ncbi.nlm.nih.gov/snp/rs1003245699) | Benign | Neutral | -0.41 | Damaging | 0.013 | Neutral | Unknown | 0.441 |
|  | [rs1010843580](https://www.ncbi.nlm.nih.gov/snp/rs1010843580) | Possibly damaging | Neutral | -0.11 | Damaging | 0.008 | Neutral | Neutral | 0.219 |
|  | [rs1023491570](https://www.ncbi.nlm.nih.gov/snp/rs1023491570) | Probably damaging | Neutral | -2.39 | Damaging | 0 | Effect | Pathogenic | 0.855 |
|  | [rs1029480822](https://www.ncbi.nlm.nih.gov/snp/rs1029480822) | Benign | Neutral | 0.08 | Tolerated | 0.521 | Neutral | Neutral | 0.206 |
|  | [rs1038896563](https://www.ncbi.nlm.nih.gov/snp/rs1038896563) | Possibly damaging | Neutral | -0.25 | Damaging | 0.002 | Neutral | Neutral | 0.235 |
|  | rs1039811463 | Benign | Neutral | -1.17 | Tolerated | 0.062 | Neutral | Unknown | 0.443 |
|  | [rs1046073933](https://www.ncbi.nlm.nih.gov/snp/rs1046073933) | Benign | Neutral | -0.75 | Tolerated | 0.087 | Effect | Pathogenic | 0.893 |
|  | [rs1050432134](https://www.ncbi.nlm.nih.gov/snp/rs1050432134) | Possibly damaging | Deleterious | -2.51 | Damaging | 0.007 | Effect | Unknown | 0.453 |
|  | [rs1054712701](https://www.ncbi.nlm.nih.gov/snp/rs1054712701) | Probably damaging | Deleterious | -3.17 | Damaging | 0.002 | Effect | Unknown | 0.66 |
|  | [rs1054712701](https://www.ncbi.nlm.nih.gov/snp/rs1054712701) | Possibly damaging | Neutral | -2.28 | Damaging | 0.007 | Neutral | Unknown | 0.289 |
|  | [rs1055108779](https://www.ncbi.nlm.nih.gov/snp/rs1055108779) | Benign | Neutral | -0.49 | Damaging | 0.025 | Neutral | Unknown | 0.424 |
|  | [rs1056005508](https://www.ncbi.nlm.nih.gov/snp/rs1056005508) | Benign | Neutral | -1.32 | Tolerated | 0.193 | Neutral | Neutral | 0.107 |
|  | [rs1056692528](https://www.ncbi.nlm.nih.gov/snp/rs1056692528) | Possibly damaging | Neutral | -1.41 | Tolerated | 0.118 | Neutral | Unknown | 0.117 |
|  | [rs1158252110](https://www.ncbi.nlm.nih.gov/snp/rs1158252110) | Possibly damaging | Deleterious | -2.58 | Damaging | 0.023 | Neutral | Unknown | 0.348 |
|  | [rs1158865993](https://www.ncbi.nlm.nih.gov/snp/rs1158865993) | Probably damaging | Deleterious | -4.22 | Damaging | 0.046 | Effect | Pathogenic | 0.906 |
|  | [rs1164029715](https://www.ncbi.nlm.nih.gov/snp/rs1164029715) | Benign | Neutral | 0.76 | Tolerated | 0.469 | Neutral | Unknown | 0.365 |
|  | [rs1168362217](https://www.ncbi.nlm.nih.gov/snp/rs1168362217) | Probably damaging | Deleterious | -2.66 | Damaging | 0.046 | Neutral | Unknown | 0.782 |
|  | [rs1169006435](https://www.ncbi.nlm.nih.gov/snp/rs1169006435) | Probably damaging | Neutral | -0.43 | Damaging | 0.007 | Effect | Neutral | 0.141 |
|  | [rs1175210435](https://www.ncbi.nlm.nih.gov/snp/rs1175210435) | Probably damaging | Deleterious | -3.82 | Damaging | 0.001 | Effect | Pathogenic | 0.893 |
|  | [rs1177378764](https://www.ncbi.nlm.nih.gov/snp/rs1177378764) | Benign | Neutral | -0.08 | Tolerated | 0.365 | Neutral | Unknown | 0.266 |
|  | [rs1183578823](https://www.ncbi.nlm.nih.gov/snp/rs1183578823) | Probably damaging | Deleterious | -7.68 | Damaging | 0 | Effect | Pathogenic | 0.939 |
|  | [rs1184039801](https://www.ncbi.nlm.nih.gov/snp/rs1184039801) | Probably damaging | Deleterious | -3.58 | Damaging | 0.019 | Effect | Pathogenic | 0.961 |
|  | [rs1188410790](https://www.ncbi.nlm.nih.gov/snp/rs1188410790) | Probably damaging | Neutral | -1.89 | Tolerated | 0.094 | Neutral | Unknown | 0.706 |
|  | [rs1190852721](https://www.ncbi.nlm.nih.gov/snp/rs1190852721) | Benign | Neutral | -0.48 | Tolerated | 0.532 | Neutral | Unknown | 0.597 |
|  | [rs1191637371](https://www.ncbi.nlm.nih.gov/snp/rs1191637371) | Probably damaging | Deleterious | -6.53 | Damaging | 0 | Effect | Pathogenic | 0.943 |
|  | [rs1192209213](https://www.ncbi.nlm.nih.gov/snp/rs1192209213) | Benign | Neutral | -0.29 | Tolerated | 0.537 | Neutral | Unknown | 0.855 |
|  | [rs1202904573](https://www.ncbi.nlm.nih.gov/snp/rs1202904573) | Probably damaging | Neutral | -2.37 | Damaging | 0.001 | Effect | Unknown | 0.718 |
|  | [rs1207268336](https://www.ncbi.nlm.nih.gov/snp/rs1207268336) | Benign | Neutral | -0.96 | Tolerated | 0.066 | Neutral | Unknown | 0.369 |
|  | [rs1212829863](https://www.ncbi.nlm.nih.gov/snp/rs1212829863) | Possibly damaging | Neutral | -1.24 | Damaging | 0.033 | Neutral | Unknown | 0.641 |
|  | [rs1215855483](https://www.ncbi.nlm.nih.gov/snp/rs1215855483) | Possibly damaging | Neutral | -1.89 | Tolerated | 0.43 | Neutral | Unknown | 0.75 |
|  | [rs1218149104](https://www.ncbi.nlm.nih.gov/snp/rs1218149104) | Possibly damaging | Neutral | -1.77 | Damaging | 0 | Effect | Unknown | 0.727 |
|  | [rs1219589831](https://www.ncbi.nlm.nih.gov/snp/rs1219589831) | Possibly damaging | Deleterious | -5.82 | Damaging | 0.001 | Effect | Pathogenic | 0.868 |
|  | [rs1221297848](https://www.ncbi.nlm.nih.gov/snp/rs1221297848) | Probably damaging | Neutral | -2.06 | Damaging | 0.001 | Neutral | Unknown | 0.278 |
|  | [rs1224001486](https://www.ncbi.nlm.nih.gov/snp/rs1224001486) | Possibly damaging | Neutral | -1.94 | Damaging | 0.042 | Effect | Unknown | 0.526 |
|  | [rs1224336230](https://www.ncbi.nlm.nih.gov/snp/rs1224336230) | Probably damaging | Deleterious | -10.36 | Damaging | 0 | Effect | Pathogenic | 0.936 |
|  | [rs1225170530](https://www.ncbi.nlm.nih.gov/snp/rs1225170530) | Benign | Neutral | 1.02 | Tolerated | 0.188 | Neutral | Unknown | 0.644 |
|  | [rs1227082843](https://www.ncbi.nlm.nih.gov/snp/rs1227082843) | Probably damaging | Neutral | -2.48 | Damaging | 0.014 | Neutral | Pathogenic | 0.944 |
|  | [rs1230115579](https://www.ncbi.nlm.nih.gov/snp/rs1230115579) | Possibly damaging | Neutral | 0.77 | Tolerated | 0.681 | Neutral | Pathogenic | 0.849 |
|  | [rs1230202192](https://www.ncbi.nlm.nih.gov/snp/rs1230202192) | Possibly damaging | Neutral | -2.22 | Damaging | 0.024 | Effect | Unknown | 0.75 |
|  | [rs1236343176](https://www.ncbi.nlm.nih.gov/snp/rs1236343176) | Benign | Neutral | -1.27 | Tolerated | 0.139 | Neutral | Unknown | 0.615 |
|  | [rs1237536573](https://www.ncbi.nlm.nih.gov/snp/rs1237536573) | Probably damaging | Neutral | -2.03 | Damaging | 0 | Effect | Unknown | 0.627 |
|  | [rs1240394269](https://www.ncbi.nlm.nih.gov/snp/rs1237536573) | Benign | Neutral | -0.34 | Tolerated | 0.248 | Neutral | Unknown | 0.251 |
|  | [rs1243599633](https://www.ncbi.nlm.nih.gov/snp/rs1243599633) | Benign | Neutral | -0.39 | Tolerated | 0.111 | Neutral | Unknown | 0.408 |
|  | [rs1244944146](https://www.ncbi.nlm.nih.gov/snp/rs1244944146) | Probably damaging | Neutral | -1.37 | Damaging | 0.011 | Effect | Pathogenic | 0.78 |
|  | [rs1250616703](https://www.ncbi.nlm.nih.gov/snp/rs1250616703) | Probably damaging | Deleterious | -7.17 | Damaging | 0 | Effect | Pathogenic | 0.881 |
|  | [rs1251042963](https://www.ncbi.nlm.nih.gov/snp/rs1251042963) | Probably damaging | Deleterious | -3.89 | Damaging | 0 | Effect | Unknown | 0.777 |
|  | [rs1254975807](https://www.ncbi.nlm.nih.gov/snp/rs1254975807) | Benign | Neutral | 0.08 | Tolerated | 0.613 | Neutral | Unknown | 0.668 |
|  | [rs1256659385](https://www.ncbi.nlm.nih.gov/snp/rs1256659385) | Benign | Neutral | -0.62 | Tolerated | 0.437 | Neutral | Neutral | 0.134 |
|  | [rs1269264458](https://www.ncbi.nlm.nih.gov/snp/rs1269264458) | Possibly damaging | Neutral | -2.05 | Damaging | 0.001 | Effect | Unknown | 0.712 |
|  | [rs1270486405](https://www.ncbi.nlm.nih.gov/snp/rs1270486405) | Probably damaging | Neutral | -1.81 | Damaging | 0.019 | Effect | Pathogenic | 0.966 |
|  | [rs1270889010](https://www.ncbi.nlm.nih.gov/snp/rs1270889010) | Probably damaging | Deleterious | -3.19 | Damaging | 0.025 | Effect | Unknown | 0.802 |
|  | [rs1273852421](https://www.ncbi.nlm.nih.gov/snp/rs1273852421) | Possibly damaging | Neutral | -0.86 | Tolerated | 0.46 | Neutral | Unknown | 0.665 |
|  | [rs1275101579](https://www.ncbi.nlm.nih.gov/snp/rs1275101579) | Benign | Neutral | -2.36 | Tolerated | 0.078 | Effect | Pathogenic | 0.954 |
|  | [rs1276375707](https://www.ncbi.nlm.nih.gov/snp/rs1276375707) | Probably damaging | Deleterious | -2.79 | Damaging | 0.001 | Effect | Unknown | 0.594 |
|  | [rs1276471970](https://www.ncbi.nlm.nih.gov/snp/rs1276471970) | Probably damaging | Deleterious | -3.72 | Damaging | 0.007 | Effect | Pathogenic | 0.868 |
|  | [rs1277076789](https://www.ncbi.nlm.nih.gov/snp/rs1277076789) | Benign | Neutral | -1.34 | Damaging | 0.049 | Effect | Unknown | 0.505 |
|  | [rs1277744858](https://www.ncbi.nlm.nih.gov/snp/rs1277744858) | Probably damaging | Neutral | -2.25 | Damaging | 0.006 | Effect | Pathogenic | 0.931 |
|  | [rs1278315350](https://www.ncbi.nlm.nih.gov/snp/rs1278315350) | Benign | Deleterious | -3.03 | Damaging | 0.003 | Effect | Unknown | 0.562 |
|  | [rs1280238984](https://www.ncbi.nlm.nih.gov/snp/rs1280238984) | Possibly damaging | Neutral | -1.97 | Damaging | 0.01 | Neutral | Unknown | 0.699 |
|  | [rs1281884581](https://www.ncbi.nlm.nih.gov/snp/rs1281884581) | Possibly damaging | Neutral | -0.95 | Damaging | 0.017 | Neutral | Unknown | 0.232 |
|  | [rs1283187808](https://www.ncbi.nlm.nih.gov/snp/rs1283187808) | Probably damaging | Neutral | -2.22 | Damaging | 0.001 | Effect | Unknown | 0.539 |
|  | [rs1285369301](https://www.ncbi.nlm.nih.gov/snp/rs1285369301) | Benign | Deleterious | -4.87 | Tolerated | 0.06 | Effect | Pathogenic | 0.926 |
|  | [rs1290398957](https://www.ncbi.nlm.nih.gov/snp/rs1290398957) | Probably damaging | Deleterious | -6.98 | Damaging | 0.002 | Effect | Pathogenic | 0.938 |
|  | [rs1290752827](https://www.ncbi.nlm.nih.gov/snp/rs1290752827) | Possibly damaging | Neutral | -1.24 | Tolerated | 0.335 | Neutral | Unknown | 0.513 |
|  | [rs1291795029](https://www.ncbi.nlm.nih.gov/snp/rs1291795029) | Benign | Deleterious | -2.72 | Damaging | 0.044 | Neutral | Unknown | 0.625 |
|  | [rs1294048735](https://www.ncbi.nlm.nih.gov/snp/rs1294048735) | Possibly damaging | Neutral | -1.89 | Damaging | 0.022 | Effect | Unknown | 0.603 |
|  | [rs1295112601](https://www.ncbi.nlm.nih.gov/snp/rs1295112601) | Possibly damaging | Deleterious | -7.34 | Damaging | 0.015 | Effect | Pathogenic | 0.89 |
|  | [rs1300216902](https://www.ncbi.nlm.nih.gov/snp/rs1300216902) | Possibly damaging | Deleterious | -4.21 | Damaging | 0.036 | Neutral | Unknown | 0.761 |
|  | [rs1302127838](https://www.ncbi.nlm.nih.gov/snp/rs1302127838) | Probably damaging | Deleterious | -3.74 | Damaging | 0.022 | Effect | Pathogenic | 0.837 |
|  | [rs1308003272](https://www.ncbi.nlm.nih.gov/snp/rs1308003272) | Benign | Neutral | -0.69 | Tolerated | 0.423 | Effect | Unknown | 0.22 |
|  | [rs1308658577](https://www.ncbi.nlm.nih.gov/snp/rs1308658577) | Possibly damaging | Deleterious | -3.14 | Damaging | 0 | Neutral | Neutral | 0.255 |
|  | [rs1315572414](https://www.ncbi.nlm.nih.gov/snp/rs1315572414) | Possibly damaging | Neutral | -1.26 | Tolerated | 0.498 | Neutral | Unknown | 0.798 |
|  | [rs1323738523](https://www.ncbi.nlm.nih.gov/snp/rs1323738523) | Benign | Neutral | 0.33 | Tolerated | 1 | Neutral | Neutral | 0.159 |
|  | [rs1330279241](https://www.ncbi.nlm.nih.gov/snp/rs1330279241) | Probably damaging | Neutral | -1.24 | Damaging | 0.047 | Neutral | Pathogenic | 0.895 |
|  | [rs1330529378](https://www.ncbi.nlm.nih.gov/snp/rs1330529378) | Possibly damaging | Deleterious | -5.44 | Damaging | 0.043 | Effect | Pathogenic | 0.826 |
|  | [rs1331241429](https://www.ncbi.nlm.nih.gov/snp/rs1331241429) | Possibly damaging | Deleterious | -2.64 | Damaging | 0.004 | Effect | Neutral | 0.219 |
|  | [rs1338895971](https://www.ncbi.nlm.nih.gov/snp/rs1338895971) | Benign | Neutral | -0.74 | Tolerated | 0.295 | Neutral | Unknown | 0.272 |
|  | [rs1339945518](https://www.ncbi.nlm.nih.gov/snp/rs1339945518) | Probably damaging | Deleterious | -2.91 | Tolerated | 0.142 | Neutral | Neutral | 0.163 |
|  | [rs1342506252](https://www.ncbi.nlm.nih.gov/snp/rs1342506252) | Probably damaging | Neutral | -1.66 | Damaging | 0 | Effect | Pathogenic | 0.898 |
|  | [rs1343377230](https://www.ncbi.nlm.nih.gov/snp/rs1343377230) | Possibly damaging | Deleterious | -5.13 | Damaging | 0.049 | Effect | Pathogenic | 0.951 |
|  | [rs1345935751](https://www.ncbi.nlm.nih.gov/snp/rs1345935751) | Benign | Neutral | 0.58 | Tolerated | 0.75 | Neutral | Unknown | 0.357 |
|  | [rs1347015690](https://www.ncbi.nlm.nih.gov/snp/rs1347015690) | Benign | Neutral | -0.23 | Tolerated | 0.959 | Neutral | Unknown | 0.236 |
|  | [rs1347150082](https://www.ncbi.nlm.nih.gov/snp/rs1347150082) | Probably damaging | Neutral | -2.44 | Damaging | 0.002 | Effect | Unknown | 0.652 |
|  | [rs1347299046](https://www.ncbi.nlm.nih.gov/snp/rs1347299046) | Possibly damaging | Neutral | -1.28 | Damaging | 0 | Neutral | neutral | 0.226 |
|  | [rs1348548231](https://www.ncbi.nlm.nih.gov/snp/rs1348548231) | Benign | Neutral | -1.6 | Tolerated | 0.053 | Neutral | Unknown | 0.258 |
|  | [rs1349469230](https://www.ncbi.nlm.nih.gov/snp/rs1349469230) | Possibly damaging | Neutral | -1.44 | Damaging | 0.003 | Neutral | Unknown | 0.603 |
|  | [rs1355061824](https://www.ncbi.nlm.nih.gov/snp/rs1355061824) | Benign | Neutral | -1.3 | Tolerated | 0.202 | Neutral | Unknown | 0.271 |
|  | [rs1357512652](https://www.ncbi.nlm.nih.gov/snp/rs1357512652) | Probably damaging | Neutral | -2.38 | Damaging | 0 | Effect | Unknown | 0.662 |
|  | [rs1359238420](https://www.ncbi.nlm.nih.gov/snp/rs1359238420) | Benign | Neutral | -0.99 | Tolerated | 0.065 | Effect | Pathogenic | 0.764 |
|  | [rs1359401489](https://www.ncbi.nlm.nih.gov/snp/rs1359401489) | Possibly damaging | Neutral | -1.34 | Tolerated | 0.289 | Neutral | Unknown | 0.276 |
|  | [rs1362466494](https://www.ncbi.nlm.nih.gov/snp/rs1362466494) | Probably damaging | Deleterious | -3.84 | Damaging | 0.002 | Effect | Pathogenic | 0.944 |
|  | [rs1362984312](https://www.ncbi.nlm.nih.gov/snp/rs1362984312) | Benign | Neutral | -0.42 | Damaging | 0.008 | Neutral | Unknown | 0.284 |
|  | [rs1363020478](https://www.ncbi.nlm.nih.gov/snp/rs1363020478) | Probably damaging | Deleterious | -3.9 | Damaging | 0.001 | Effect | Unknown | 0.552 |
|  | [rs1366122974](https://www.ncbi.nlm.nih.gov/snp/rs1366122974) | Possibly damaging | Deleterious | -2.99 | Tolerated | 0.098 | Effect | Pathogenic | 0.903 |
|  | [rs1366123623](https://www.ncbi.nlm.nih.gov/snp/rs1366123623) | Possibly damaging | Neutral | -2.13 | Tolerated | 0.278 | Effect | Neutral | 0.222 |
|  | [rs1366555549](https://www.ncbi.nlm.nih.gov/snp/rs1366555549) | Probably damaging | Deleterious | -3.28 | Tolerated | 0.059 | Effect | Unknown | 0.917 |
|  | [rs1371304452](https://www.ncbi.nlm.nih.gov/snp/rs1371304452) | Benign | Neutral | -0.68 | Tolerated | 0.41 | Neutral | Neutral | 0.263 |
|  | [rs1372909550](https://www.ncbi.nlm.nih.gov/snp/rs1372909550) | Probably damaging | Deleterious | -5.25 | Damaging | 0.001 | Effect | Unknown | 0.745 |
|  | [rs1374396490](https://www.ncbi.nlm.nih.gov/snp/rs1374396490) | Benign | Neutral | -2.09 | Damaging | 0.045 | Neutral | Unknown | 0.518 |
|  | [rs1375575897](https://www.ncbi.nlm.nih.gov/snp/rs1375575897) | Probably damaging | Deleterious | -4.02 | Damaging | 0.001 | Effect | Pathogenic | 0.91 |
|  | [rs1378829324](https://www.ncbi.nlm.nih.gov/snp/rs1378829324) | Benign | Deleterious | -2.54 | Damaging | 0.007 | Neutral | Unknown | 0.343 |
|  | [rs1380696549](https://www.ncbi.nlm.nih.gov/snp/rs1380696549) | Probably damaging | Deleterious | -3.21 | Tolerated | 0.136 | Effect | Unknown | 0.67 |
|  | [rs1383735594](https://www.ncbi.nlm.nih.gov/snp/rs1383735594) | Possibly damaging | Neutral | -1.57 | Tolerated | 0.178 | Neutral | Pathogenic | 0.853 |
|  | [rs1383948441](https://www.ncbi.nlm.nih.gov/snp/rs1383948441) | Probably damaging | Deleterious | -3.86 | Damaging | 0.001 | Effect | Pathogenic | 0.933 |
|  | [rs1384054598](https://www.ncbi.nlm.nih.gov/snp/rs1384054598) | Possibly damaging | Neutral | -1.37 | Tolerated | 0.191 | Neutral | Unknown | 0.58 |
|  | [rs1385939134](https://www.ncbi.nlm.nih.gov/snp/rs1385939134) | Possibly damaging | Neutral | -2.09 | Damaging | 0.001 | Effect | Pathogenic | 0.853 |
|  | [rs1386656928](https://www.ncbi.nlm.nih.gov/snp/rs1386656928) | Possibly damaging | Neutral | -0.56 | Tolerated | 0.063 | Effect | Unknown | 0.688 |
|  | [rs1391902915](https://www.ncbi.nlm.nih.gov/snp/rs1391902915) | Possibly damaging | Neutral | -0.23 | Tolerated | 0.136 | Effect | Neutral | 0.167 |
|  | [rs1394481073](https://www.ncbi.nlm.nih.gov/snp/rs1394481073) | Benign | Neutral | -0.05 | Tolerated | 0.725 | Neutral | Neutral | 0.184 |
|  | [rs1394757420](https://www.ncbi.nlm.nih.gov/snp/rs1394757420) | Probably damaging | Deleterious | -5.07 | Damaging | 0.002 | Effect | Pathogenic | 0.909 |
|  | [rs1397056325](https://www.ncbi.nlm.nih.gov/snp/rs1397056325) | Probably damaging | Neutral | -0.94 | Damaging | 0.011 | Neutral | Unknown | 0.677 |
|  | [rs1400180568](https://www.ncbi.nlm.nih.gov/snp/rs1400180568) | Benign | Neutral | -1.68 | Tolerated | 0.092 | Neutral | Unknown | 0.486 |
|  | [rs1402486267](https://www.ncbi.nlm.nih.gov/snp/rs1402486267) | Benign | Neutral | -1.91 | Damaging | 0.015 | Neutral | Neutral | 0.076 |
|  | [rs1403341767](https://www.ncbi.nlm.nih.gov/snp/rs1403341767) | Benign | Deleterious | -2.69 | Damaging | 0.001 | Effect | Unknown | 0.749 |
|  | [rs1406288053](https://www.ncbi.nlm.nih.gov/snp/rs1406288053) | Possibly damaging | Neutral | -1.95 | Damaging | 0.036 | Effect | Unknown | 0.513 |
|  | [rs1407370674](https://www.ncbi.nlm.nih.gov/snp/rs1407370674) | Benign | Neutral | -0.58 | Damaging | 0.011 | Effect | Unknown | 0.241 |
|  | [rs1416091793](https://www.ncbi.nlm.nih.gov/snp/rs1416091793) | Benign | Neutral | 0.55 | Tolerated | 1 | Neutral | Unknown | 0.083 |
|  | [rs1419544157](https://www.ncbi.nlm.nih.gov/snp/rs1419544157) | Benign | Neutral | -1.14 | Tolerated | 0.078 | Neutral | Unknown | 0.415 |
|  | [rs1423083583](https://www.ncbi.nlm.nih.gov/snp/rs1423083583) | Probably damaging | Deleterious | -2.94 | Damaging | 0 | Effect | Unknown | 0.471 |
|  | [rs1425319727](https://www.ncbi.nlm.nih.gov/snp/rs1425319727) | Probably damaging | Deleterious | -3.28 | Damaging | 0.001 | Effect | Unknown | 0.474 |
|  | [rs1428334171](https://www.ncbi.nlm.nih.gov/snp/rs1428334171) | Probably damaging | Deleterious | -3.29 | Damaging | 0 | Effect | Pathogenic | 0.863 |
|  | [rs1433376279](https://www.ncbi.nlm.nih.gov/snp/rs1433376279) | Possibly damaging | Deleterious | -2.89 | Tolerated | 0.059 | Effect | Pathogenic | 0.868 |
|  | [rs1436939063](https://www.ncbi.nlm.nih.gov/snp/rs1436939063) | Probably damaging | Deleterious | -4.01 | Damaging | 0 | Effect | Pathogenic | 0.958 |
|  | [rs1438024604](https://www.ncbi.nlm.nih.gov/snp/rs1438024604) | Probably damaging | Deleterious | -5.29 | Damaging | 0 | Effect | Unknown | 0.729 |
|  | [rs1438512681](https://www.ncbi.nlm.nih.gov/snp/rs1438512681) | Possibly damaging | Neutral | -2.21 | Tolerated | 0.111 | Neutral | Pathogenic | 0.965 |
|  | [rs1441263206](https://www.ncbi.nlm.nih.gov/snp/rs1441263206) | Possibly damaging | Deleterious | -3.29 | Damaging | 0.019 | Neutral | Unknown | 0.667 |
|  | [rs1442230907](https://www.ncbi.nlm.nih.gov/snp/rs1442230907) | Benign | Neutral | -1.05 | Tolerated | 0.074 | Neutral | Unknown | 0.683 |
|  | [rs1445779721](https://www.ncbi.nlm.nih.gov/snp/rs1445779721) | Probably damaging | Deleterious | -5.2 | Damaging | 0.015 | Effect | Pathogenic | 0.885 |
|  | [rs1447075960](https://www.ncbi.nlm.nih.gov/snp/rs1447075960) | Probably damaging | Neutral | -0.44 | Damaging | 0.037 | Neutral | Unknown | 0.348 |
|  | [rs1447805795](https://www.ncbi.nlm.nih.gov/snp/rs1447805795) | Possibly damaging | Deleterious | -2.95 | Damaging | 0.003 | Effect | Pathogenic | 0.868 |
|  | [rs1448096881](https://www.ncbi.nlm.nih.gov/snp/rs1448096881) | Possibly damaging | Neutral | -0.98 | Damaging | 0.005 | Neutral | Unknown | 0.515 |
|  | [rs1452002022](https://www.ncbi.nlm.nih.gov/snp/rs1452002022) | Possibly damaging | Neutral | -1.92 | Damaging | 0.001 | Effect | Pathogenic | 0.818 |
|  | [rs1452181228](https://www.ncbi.nlm.nih.gov/snp/rs1452181228) | Probably damaging | Neutral | -1.41 | Damaging | 0.006 | Neutral | Unknown | 0.64 |
|  | [rs1458512225](https://www.ncbi.nlm.nih.gov/snp/rs1458512225) | Benign | Deleterious | -3.39 | Damaging | 0.023 | Effect | Unknown | 0.794 |
|  | [rs1459605752](https://www.ncbi.nlm.nih.gov/snp/rs1459605752) | Possibly damaging | Deleterious | -2.96 | Damaging | 0.013 | Effect | Pathogenic | 0.878 |
|  | [rs1461838608](https://www.ncbi.nlm.nih.gov/snp/rs1461838608) | Probably damaging | Neutral | -0.49 | Tolerated | 0.942 | Neutral | Unknown | 0.288 |
|  | [rs1463421426](https://www.ncbi.nlm.nih.gov/snp/rs1463421426) | Probably damaging | Deleterious | -2.74 | Damaging | 0.009 | Neutral | Pathogenic | 0.882 |
|  | [rs1464232313](https://www.ncbi.nlm.nih.gov/snp/rs1464232313) | Probably damaging | Deleterious | -4.04 | Damaging | 0.012 | Neutral | Pathogenic | 0.917 |
|  | [rs1468750371](https://www.ncbi.nlm.nih.gov/snp/rs1468750371) | Probably damaging | Neutral | -2.08 | Damaging | 0.003 | Neutral | Pathogenic | 0.874 |
|  | [rs1476893897](https://www.ncbi.nlm.nih.gov/snp/rs1476893897) | Probably damaging | Deleterious | -5.48 | Damaging | 0 | Effect | Unknown | 0.799 |
|  | [rs1476956484](https://www.ncbi.nlm.nih.gov/snp/rs1476956484) | Probably damaging | Neutral | -1.49 | Damaging | 0.013 | Neutral | Unknown | 0.607 |
|  | [rs1480440632](https://www.ncbi.nlm.nih.gov/snp/rs1480440632) | Probably damaging | Neutral | -1.33 | Tolerated | 0.102 | Neutral | Unknown | 0.655 |
|  | [rs1483420106](https://www.ncbi.nlm.nih.gov/snp/rs1483420106) | Possibly damaging | Neutral | -0.38 | Tolerated | 0.519 | Neutral | Neutral | 0.066 |
|  | [rs1563044670](https://www.ncbi.nlm.nih.gov/snp/rs1563044670) | Possibly damaging | Neutral | 0.12 | Damaging | 0.006 | Neutral | Unknown | 0.346 |
|  | [rs1563046071](https://www.ncbi.nlm.nih.gov/snp/rs1563046071) | Probably damaging | Neutral | -1.79 | Damaging | 0 | Neutral | Unknown | 0.566 |
|  | [rs1563054003](https://www.ncbi.nlm.nih.gov/snp/rs1563054003) | Possibly damaging | Neutral | -1.52 | Tolerated | 0.071 | Effect | Unknown | 0.636 |
|  | [rs1563061750](https://www.ncbi.nlm.nih.gov/snp/rs1563061750) | Probably damaging | Deleterious | -6.87 | Damaging | 0 | Effect | Pathogenic | 0.94 |
|  | [rs1563064369](https://www.ncbi.nlm.nih.gov/snp/rs1563064369) | Possibly damaging | Deleterious | -2.64 | Tolerated | 0.124 | Neutral | Unknown | 0.759 |
|  | [rs1563064499](https://www.ncbi.nlm.nih.gov/snp/rs1563064499) | Possibly damaging | Deleterious | -3.03 | Tolerated | 0.083 | Effect | Unknown | 0.493 |
|  | [rs1584971788](https://www.ncbi.nlm.nih.gov/snp/rs1584971788) | Possibly damaging | Deleterious | -2.73 | Damaging | 0.008 | Effect | Unknown | 0.721 |
|  | [rs1584983348](https://www.ncbi.nlm.nih.gov/snp/rs1584983348) | Possibly damaging | Neutral | -2.02 | Damaging | 0.014 | Effect | Pathogenic | 0.868 |
|  | [rs1584983574](https://www.ncbi.nlm.nih.gov/snp/rs1584983574) | Probably damaging | Neutral | -1.89 | Damaging | 0.048 | Effect | Unknown | 0.083 |
|  | [rs1585001758](https://www.ncbi.nlm.nih.gov/snp/rs1585001758) | Probably damaging | Deleterious | -3.67 | Damaging | 0.045 | Neutral | Unknown | 0.64 |
|  | [rs1585001783](https://www.ncbi.nlm.nih.gov/snp/rs1585001783) | Benign | Neutral | -0.56 | Tolerated | 0.198 | Neutral | Unknown | 0.627 |
|  | [rs1585001804](https://www.ncbi.nlm.nih.gov/snp/rs1585001804) | Possibly damaging | Deleterious | -3.31 | Tolerated | 0.086 | Effect | Pathogenic | 0.91 |
