# Supplementary material for: A comprehensive in silico analysis of the deleterious nonsynonymous SNPs of human FOXP2 protein
Source: PLoS One. 2022 Aug 9;17(8):e0272625. doi: 10.1371/journal.pone.0272625 (PMC9362936; doi:10.1371/journal.pone.0272625)
Supplement: S3 Table — (DOCX) [file pone.0272625.s004.docx]

| **Interacting residues** | **Distance** | **Bond type** | **Interacting residues** | **Distance** | **Bond type** | **Interacting residues** | **Distance** | **Bond type** | **Interacting residues** | **Distance** | **Bond type** | **Interacting residues** | **Distance** | **Bond type** |
| --- | --- | --- | --- | --- | --- | --- | --- | --- | --- | --- | --- | --- | --- | --- |
| **Wild type** |  |  | **L558P** |  |  | **R536G** |  |  | **R553C** |  |  | **Y531H** |  |  |
| ARG 504 | 4.667 | AC | ARG 504 | 5.376 | AC | ARG 504 | 5.077 | AC | ARG 504 | 5.379 | AC | ARG 504 | 4.335 | AC |
| TYR 509 | 2.471 | CoHB | TYR 509 | 2.916 | CoHB | TYR 509 | 2.599 | CoHB | TYR 509 | 2.374 | CoHB | TYR 509 | 2.671 | CoHB |
| HIS 554 | 2.900 | CHB | THR 547 | 3.334 | CHB | HIS 554 | 2.914 | CHB | HIS 554 | 2.838 | CHB | THR 547 | 2.964 | CHB |
| LEU 558 | 3.609 | PS | PRO 558 | 4.723 | PAl | LEU 558 | 3.369 | PS | PHE 541 | 4.717 | PAn | PRO 505 | 3.406 | CHB |
| PRO 505 | 3.165 | CHB | HIS 559 | 3.753 | PDHB | HIS 559 | 4.161 | PDHB | HIS 559 | 4.149 | PDHB | HIS 554 | 2.923 | CHB |
| TRP 548 | 4.050 | PAn |  |  |  |  |  |  | LEU 558 | 3.406 | PS | LEU 558 | 3.602 | PS |
| ALA 551 | 5.446 | PAl |  |  |  |  |  |  |  |  |  | TRP 548 | 4.375 | PAn |
|  |  |  |  |  |  |  |  |  |  |  |  |  |  |  |

**Interactions of amino acid residues with native and mutant FoxP2 protein**

AC= Attractive Charge, CoHB= Conventional Hydrogen Bond, CHB=Carbon Hydrogen Bond, PS=Pi sigma, PDHB= Pi-Donor Hydrogen Bond, PAn=Pi-Anion, PAl=Pi-Alkyl
